# Graft-Versus-Host Disease Prevention by *In Vitro*-Generated Myeloid-Derived Suppressor Cells Is Exclusively Mediated by the CD11b+CD11c+ MDSC Subpopulation

**DOI:** 10.3389/fimmu.2021.754316

**Published:** 2021-10-14

**Authors:** Jasmin Scheurer, Kerstin Kitt, Heinrich J. Huber, Katrin Fundel-Clemens, Stefan Pflanz, Klaus-Michael Debatin, Gudrun Strauss

**Affiliations:** ^1^ Department of Pediatrics and Adolescent Medicine, University Medical Center Ulm, Ulm, Germany; ^2^ Department of Cancer Immunology and Immune Modulation, Boehringer Ingelheim Pharma Co KG, Biberach an der Riss, Germany; ^3^ Global Computational Biology and Digital Sciences, Boehringer Ingelheim Pharma Co KG, Biberach an der Riss, Germany; ^4^ Drug Discovery Services, Boehringer Ingelheim Regional Center Vienna (RCV) GmbH & Co KG, Vienna, Austria

**Keywords:** graft- *versus* -host disease, prophylaxis, myeloid-derived suppressor cells, allogeneic bone marrow transplantation, mouse model, type 2 immune response, GVT effect

## Abstract

Myeloid-derived suppressor cells (MDSCs) are a heterogeneous population of myeloid progenitor cells that dampen overwhelming adaptive immune responses through multiple mechanisms and are recognized as an attractive novel immune intervention therapy for counteracting the destructive effects of graft- *versus* -host disease (GVHD) developing after allogeneic bone marrow transplantation (BMT). MDSCs can be produced in great numbers for cellular therapy, but they present a mixture of subsets whose functions in GVHD prevention are undefined. Here, we generated MDSCs *in vitro* from murine BM cells in the presence of GM-CSF and defined the integrin CD11c as a marker to subdivide MDSCs into two functional subgroups: CD11b+CD11c+ and CD11b+CD11c− MDSCs. Isolated CD11b+CD11c+ and CD11b+CD11c− MDSCs both inhibited alloantigen-stimulated T-cell proliferation *in vitro*, although CD11b+CD11c+ MDSCs were more efficient and expressed higher levels of different immunosuppressive molecules. Likewise, expression of surface markers such as MHC class II, CD80, CD86, or PD-L1 further delineated both subsets. Most importantly, only the adoptive transfer of CD11b+CD11c+ MDSCs into a single MHC class I-disparate allogeneic BMT model prevented GVHD development and strongly decreased disease-induced mortality, while CD11b+CD11c− MDSCs were totally ineffective. Surprisingly, allogeneic T-cell homing and expansion in lymphatic and GVHD target organs were not affected by cotransplanted CD11b+CD11c+ MDSCs indicating a clear contradiction between *in vitro* and *in vivo* functions of MDSCs. However, CD11b+CD11c+ MDSCs shifted immune responses towards type 2 immunity reflected by increased Th2-specific cytokine expression of allogeneic T cells. Induction of type 2 immunity was mandatory for GVHD prevention, since CD11b+CD11c+ MDSCs were ineffective if recipients were reconstituted with STAT6-deficient T cells unable to differentiate into Th2 cells. Most importantly, the beneficial graft- *versus* -tumor (GVT) effect was maintained in the presence of CD11b+CD11c+ MDSCs since syngeneic tumor cells were efficiently eradicated. Strong differences in the transcriptomic landscape of both subpopulations underlined their functional differences. Defining CD11b+CD11c+ MDSCs as the subset of *in vitro*-generated MDSCs able to inhibit GVHD development might help to increase efficiency of MDSC therapy and to further delineate relevant target molecules and signaling pathways responsible for GVHD prevention.

## Introduction

In the year 2007, myeloid-derived suppressor cells (MDSCs) were introduced as a heterogeneous population of myeloid progenitors with potent immunosuppressive functions (1, 2) that expand under various inflammatory pathological conditions such as chronic inflammation, autoimmune diseases, infections, and cancer. Under inflammatory conditions, MDSCs fail to complete their regular differentiation into mature macrophages, granulocytes, or dendritic cells and are phenotypically and functionally distinct from normal myeloid cells. T cells are the preferred and major targets of MDSCs. MDSCs inhibit T-cell responses by versatile mechanisms including nutrient depletion, nitrosylation, apoptosis, or blockade of lymphocyte homing or induction of Tregs (3, 4). By studying the role of MDSCs in different disease entities, it turned out that they strongly contribute to the decision whether immune responses develop towards type 1 or type 2 immunity. MDSCs shift the balance towards Th2 immunity in pathologies such as sepsis, viral infections, or certain types of cancers (5–8), while they support Th1 immunity in Th2-driven asthma-related airway inflammation (9, 10).

Considering their immunomodulatory functions, MDSCs represent attractive candidates to counterbalance overwhelming immune responses associated with T-cell-mediated diseases. Graft- *versus* -host disease (GVHD) represents a disease which develops after allogeneic bone marrow (BMT) transplantation and is induced by activation and expansion of alloantigen-activated mature transplant-derived T cells. These donor T cells attack and destroy recipient tissue finally leading to life-threatening posttransplantation complications, which dramatically limit the success of allogeneic stem cell transplantation for treatment of hematological malignancies and genetic disorders (11). However, transplanted allogeneic T cells also mediate the graft- *versus* -tumor effect (GVT), which ensures eradication of alloantigen expressing residual tumor cells. Therefore, GVHD treatment strategies aiming to interfere with allogenic T-cell activation, proliferation, and function should be balanced in terms to prevent allogeneic T-cell-mediated tissue destruction while simultaneously guaranteeing efficient T-cell immunity to cope with infections and destroy residual tumor cells (12).

MDSCs for adoptive cell therapy of GVHD can be successfully generated in mice. MDSCs can be directly isolated from tumor-bearing mice (13) or after *in vivo* administration of 3′5′-cytidylylguanosine (CpG), granulocyte-colony stimulating factor (G-CSF), or a synthetic G-CSF/Flt-3 ligand. Subsequent adoptive transfer of the isolated MDSCs in allogeneic BM recipients efficiently prevents GVHD development (13–16). Different precursor cells and cytokine combinations are suitable for *in vitro* induction and expansion of MDSCs. While Zhou et al. used mouse embryonic stem cells activated with a mixture of cytokines in a three-step differentiation strategy (17), MDSCs currently are mostly induced by culturing unseparated BM cells in GM-CSF alone or in combinations with cytokines such as G-CSF or IL-13 (18–20). Independent of the cytokine combination used, adoptive transfer of *in vitro*-generated MDSCs efficiently prevents GVHD induction, while tumor reactivity in MDSC-treated mice is maintained. Although randomized trials proving therapeutic potential of MDSCs in humans are lacking, promising results are obtained from humanized mouse models receiving *in vitro*-expanded human MDSCs for prevention of xenogeneic GVHD (21, 22).

MDSCs either isolated *ex vivo* from tissues or generated *in vitro* from hematopoietic precursor cells always present a mixture of cells. Classically, unseparated murine MDSCs coexpress CD11b and Gr-1 and expression of Ly-6C and Ly-6G further subdivides MDSCs into the two major subpopulations: monocytic (M) MDSCs (CD11b^+^Ly-6G^-^Ly-6C^high^) and polymorphonuclear (PMN) MDSCs (CD11b^+^Ly-6G^-^Ly-6C^high^) (23, 24). Since Ly-6G and Ly-6C are also expressed on differentiated monocytes and mature neutrophils, further marker panels have been designed including transcription factors, cytokines, and effector molecules to distinguish MDSC subsets from fully matured myeloid cells (25), but the final identification as MDSCs is always designated by their ability to mediate T-cell suppressive functions (26, 27).

Currently, it is not defined which subset of MDSCs contribute to immunosuppression and GVHD prevention in the context of allogeneic BMT. In recent work, we could show that adoptive transfer of CD11b+Gr-1+ MDSCs induced from BM cells in the presence of GM-CSF efficiently prevented GVHD development in two different allogeneic BMT models. GVHD inhibition was mostly attributed to the ability of MDSCs to shift the immune response in the transplanted recipients towards type 2 immunity (18). Interestingly, phenotypic characterization of the *in vitro*-generated MDSCs indicated that these cells were not a unique population but could be distinguished by the expression of the integrin CD11c. CD11b+CD11c+ MDSC subpopulations exhibited increased expression of CD301b, which expression is linked to the Th2-inducing abilities of DCs (28). Additionally, transcription factors IRF4 and Klf4 also associated with Th2 induction (29, 30) were upregulated compared with CD11b+CD11c− MDSCs indicating that both subpopulations might exhibit different properties in GVHD prevention. In the current study, we therefore aimed to further characterize CD11b+CD11c+ and CD11b+CD11c− MDSCs for their T-cell suppressive capacities and their function in GVHD prevention. Although both subsets suppressed T-cell proliferation *in vitro*, only CD11b+CD11c+ MDSCs prevented GVHD development after allogeneic BMT while maintaining tumor cytotoxicity. GVHD prevention was totally dependent on the ability of CD11b+CD11c+ MDSCs to shift the immune response towards type 2 immunity. Strong differences in the transcriptomic landscape of both MDSC subsets further underlined their functional differences and might be used in further studies to delineate molecules and pathways responsible for MDSC-mediated GVHD inhibition.

## Material and Methods

### Tissue Preparation

#### Bone Marrow

Bone marrow (BM) cells were isolated with 26-gauge needle from femurs and tibias. Single-cell suspensions were prepared using a syringe with 20-gauge needle, and erythrocytes were depleted.

#### Spleen

Splenic single-cell suspensions were prepared by pouring the spleen through a 70-µm cell strainer followed by erythrocyte depletion.

#### Liver

Liver was perfused by the injection of 5 ml liver perfusion medium (Gibco, Carlsbad, CA, USA), followed by 5 ml liver digest medium (Gibco) into the vena cava inferior. Without the gall bladder, liver was digested for 30 min at 37°C in 10 ml liver digest medium. Single-cell suspensions were prepared by pouring the liver through a 70-µm cell strainer. Liver cells were suspended in 35% Percoll (Sigma-Aldrich, St. Louis, MO, USA), followed by overlaying cells onto 70% Percoll. The gradient was centrifuged at 2,000 rpm for 20 min. Interfaces containing liver leukocytes were collected, and residual erythrocytes were depleted.

#### Serum

Serum was collected from submandibular blood. Serum was stored at −80°C in cytokine stabilization buffer (U-CyTech Biosciences, Utrecht, Netherlands) (1:20 of collected serum volume) until ProcartaPlex Multiplex immunoassays (ThermoFisher Scientific, Waltham, MA, USA) were performed.

### MDSC *In Vitro* Generation

MDSCs were generated *in vitro* by incubating freshly isolated BM cells with 250 U/ml murine GM-CSF for 4 days at 37°C in an atmosphere with 7.5% CO_2_. BM cells at 9 × 10^6^–1 × 10^7^ were cultured in α-minimum essential medium (Lonza, Basel, Switzerland), 10% fetal calf serum (Sigma Aldrich), 2 mM l-glutamine (Gibco), 1 mM sodium-pyruvate (Gibco), 100 U/ml penicillin-streptomycin (Gibco), and 0.05 mM 2-mercaptoethanol (Gibco) in Ø 15 cm culture dishes “Cell+” (Sarstedt, Germany).

### Isolation of CD11b+CD11c+ and CD11b+CD11c− MDSCs

CD11b+CD11c+ MDSCs were positively isolated by magnetic-activated cell sorting using anti-CD11c MicroBeads (Miltenyi, Bergisch Gladbach, Germany) according to manufacturer’s protocol. CD11b+CD11c- MDSCs were isolated from the flow-through of CD11c isolation by loading the flow-through on a depleting LD column (Miltenyi). Purity of both MDSC subpopulations ranged between 85% and 99%.

### Isolation of CD3+ T Cells

CD3+ T cells were positively isolated from splenic single-cell suspensions by magnetic-activated cell sorting using the CD3ϵ MicroBead Kit (Miltenyi) according to manufacturer’s protocol. Purity of isolated T cells was over 70%.

### Mice and Bone Marrow Transplantation

#### Mice

Mouse strains used are listed in **Supplementary Table S1**.

#### BMT

One day before BMT, B6.bm1 recipient mice received total body irradiation with 12 Gy split in two doses 3 h apart from a ^137^Cs source. BM cells were depleted from T cells as described previously (18, 19). Mice were intravenously reconstituted with 5 × 10^6^ T-cell-depleted BM (TCD-BM) in the presence or absence of 2 × 10^7.^spleen cells (SC). *In vitro*-generated CD11b+CD11c+ or CD11b+CD11c− MDSCs at 1 × 10^6^ were coinjected with the transplant. In studies analyzing the GVT effect 5 × 10^4^ JM6 thymoma (18) were coinjected with the transplant. Clinical GVHD was evaluated according to Cooke et al. (31) by evaluating the parameters weight loss, activity, posture, fur texture, and skin integrity. Animals euthanized during the experiment due to their moribund state remained included in the calculation until the end of experiment with their final GVHD scores. All animal experiments were performed according to the international regulations for the care and use of laboratory animals and were approved by the local ethical committee Regierungspräsidium Tübingen, Germany.

### Carboxyfluorescein Diacetate Succinimidyl Ester Labeling

Cells at 2 × 10^7^ in 10 ml PBS containing 5% FCS were labeled with 50 µM carboxyfluorescein diacetate succinimidyl ester (CFSE) (ThermoFisher Scientific, MA, USA) for 10 min at 37°C in the dark.

### Mixed Lymphocyte Reaction

CFSE-labeled B6.SJL-derived SCs at 2.5 × 10^5^ were stimulated with 2.5 × 10^5^ irradiated (33 Gy) DBA/2-derived SCs in the absence or presence of B6-derived CD11b+CD11c+ or CD11b+CD11c− MDSCs. iNOS was inhibited using 500 µM L-N^G^-monomethyl-arginine-citrate (L-NMMA) (Merck, Darmstadt, Germany) and PD-L2 was blocked using 10 μg/ml antimouse PD-L2 antibodies (Biocell, St. Irvine, CA, USA). Mixed lymphocyte reactions (MLRs) were cultured in α-MEM medium (Lonza) supplemented with 10% FCS (Sigma Aldrich), 2 mM l-glutamine (Gibco), 1 mM sodium-pyruvate (Gibco), 100 U/ml penicillin-streptomycin (Gibco), and 0.05 mM 2-mercaptoethanol (Gibco) for 4 days at 37°C in an atmosphere with 7.5% CO_2_. After 4 days, T-cell proliferation was determined using flow cytometry and percentage of T-cell suppression was calculated.

### Flow Cytometry

Cells at 5 × 10^5^–1 × 10^6^ were stained with respective fluorochrome-conjugated antibodies. Antibodies used are listed in **Supplementary Table S2**. Flow cytometric analyses were performed on a LSR II flow cytometer (BD Biosciences, Franklin Lakes, NJ, USA).

### Quantitative Reverse-Transcription Polymerase Chain reaction

Quantitative reverse-transcription polymerase chain reaction (qRT-PCR) was performed using the SsoAdvanced™ Universal SYBR^®^ Green Supermix (BIO-RAD, Irvine, CA, USA) and analysis was performed on a CFX Connect Optics Module (BIO-RAD). Relative expression was determined using the comparative C_T_ method. Mouse aryl hydrocarbon receptor-interacting protein (AIP) was used as a housekeeping gene. Primer sets used are listed in **Supplementary Table S3**.

### Cytokine Analysis Using ProcartaPlex™ Multiplex Immunoassay

Cytokine concentrations of 12.5 µl blood serum or 25 µl cell culture supernatant were analyzed by ProcartaPlex™ multiplex immunoassays (ThermoFisher Scientific) according to manufacturer’s protocol. Analyses were performed on a BIO-RAD Bioplex 200 system (BIO-RAD).

### RNA Isolation and Quality Control

FACS-sorted CD11b+CD11c+ and CD11b+CD11c− MDSCs were collected in RLT buffer (QIAGEN, Hilden, Germany) supplemented with 2-mercaptoethanol followed by RNA extraction using the RNeasy Mini Kit (Qiagen) and adding an on-column DNA digestion step according to manufacturer’s instructions. Total RNA was quantitatively and qualitatively assessed using the absorbance-based Take3 microvolume plate system on a Cytation 5 instrument (BioTek, Bad Friedrichshall, Germany) and the Standard Sensitivity RNA Analysis DNF-471 Kit on a 12-channel Fragment Analyzer (Agilent Technologies, Santa Clara, CA, USA), respectively. Concentrations averaged at 310 ng/µl while RIN values ranged from 8.6 to 10, with an average of 9.8.

### Whole Transcriptome Profiling With PolyA Enrichment (mRNA-Seq)

MDSC-derived RNA samples were normalized, and a RNA input of 100 ng was used for library construction with the NEBNext Ultra II Directional RNA Library Prep Kit for Illumina #E7760, together with the NEBNext Poly(A) mRNA Magnetic Isolation Module #E7490 upstream and the NEXNext Multiplex Oligos for Illumina #E7600 downstream (New England Biolabs, Frankfurt am Main, Germany). Ampure XP beads (Beckman Coulter, Brea, CA, USA) were used for double-stranded cDNA purification. mRNA sequencing libraries were quantified by the High Sensitivity dsDNA Quanti-iT Assay Kit (ThermoFisher Scientific) on a Synergy HTX (BioTek). Library molarity averaged at 134 nM. Final library size distribution was assessed (smear analysis of 364 bp average and adapter dimer presence <0.5%) by the High Sensitivity Small Fragment DNF-477 Kit on a 12-channel Fragment Analyzer (Agilent Technologies). All sequencing libraries passed quality check, were normalized, pooled, and spiked in with PhiX Control v3 (Illumina, San Diego, CA, USA). The library pool was subsequently clustered with the HiSeq 3000/4000 SR Cluster Kit on a cBot and sequenced on a HiSeq 3000 Sequencing System (Illumina) with single index, single read at 85 bp length (Read parameters: Rd1: 85, Rd2: 8), reaching an average depth of 29 million Pass-Filter reads per sample (11% CV).

### mRNA-Seq Computational Analysis

Illumina reads were converted to FASTQ files and aligned to the mouse reference genomes from Ensembl 70 (http://www.ensembl.org) using the STAR v2.5.2 program on default settings (32). SAM files were converted by samtools v0.1.18 (33) to BAM files. Sequenced read quality was checked with FastQC v0.11.2 (http://www.bioinformatics.babraham.ac.uk/projects/fastqc/), and alignment quality metrics were calculated using the RNASeQC v1.1.8 (34). Duplication rates were assessed with bamUtil v1.0.11 (35) and dupRadar v1.4 (36). Gene expression levels were quantified by Cufflinks v2.2.1 (37) to get reads per kilobase per million mapped reads (RPKM) as well as FeatureCounts (38) to get read counts. Differential expression analysis was performed based on voom-normalized (39) read counts as input for the Bioconductor R package LIMMA (40). The batch number was used as a factor in the LIMMA linear regression model. *p*-values were corrected for multiple testing by Benjamini–Hochberg. Complete mRNA-sequencing (mRNA-Seq) data are available at Gene Expression Omnibus (GEO accession number: GSE182262).

### Statistics

Data were analyzed using Mann-Whitney *U* test or unpaired Student’s *t*-test. For multiple comparisons ANOVA Tukey multiple comparison test or Kruskal-Wallis test were used. Survival studies were analyzed using Log-Rank (Mantel-Cox) test. Results were considered significant if *p* < 0.05. Statistical tests were performed with GraphPad Prism 8.

## Results

### Expression of CD11b and CD11c Distinguishes Two Subpopulations of *In Vitro*-Generated MDSCs

**Figure 1:**
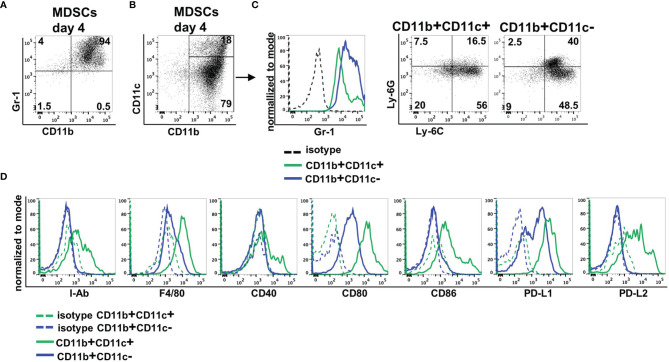
*In vitro*-generated MDSCs consist of CD11b+CD11c+ and CD11b+CD11c− MDSCs. MDSCs were generated from BM cells in the presence of GM-CSF. **(A)** After 4 days, MDSCs were stained for CD11b and Gr-1 expression or **(B)** for CD11b and CD11c expression. **(C)** Expression of Gr-1 was determined on CD11b+CD11c+ and CD11b+CD11c− MDSCs, and CD11b+CD11c+ and CD11b+ and CD11c+ MDSCs were designated to M-MDSCs or PMN-MDSCs by staining Ly-6C and Ly-6G. **(D)** Staining of various surface markers to define differences in the expression on CD11b+CD11c+ and CD11b+CD11c− MDSCs. FACS diagrams show one representative experiment out of at least three experiments performed.

MDSCs were generated from BM cells in the presence of GM-CSF. After 4 days, more than 90% of cells expressed CD11b and Gr-1 indicative for successful MDSC generation *in vitro* (**Figure 1A**). By staining CD11b and CD11c *in vitro*-generated MDSCs could be separated into two major subpopulations. Eighty percent of MDSCs exhibited solely CD11b positivity, while 20% coexpressed CD11b and CD11c (**Figure 1B**). CD11b+CD11c+ MDSCs could be distinguished from CD11b+CD11c− MDSCs by decreased Gr-1 expression. To assign both MDSC subsets to M-MDSCs (Ly-6C^high^Ly6G^−^) or PMN-MDSCs (Ly-6C^low^Ly6G^+^), we costained for Ly-6C and Ly-6G. CD11b+CD11c+ MDSCs consist of about 60% M-MDSCs and low percentage of PMN-MDSCs, while 20% of the cells neither expresses Ly-6G and Ly-6C. CD11b+CD11c− MDSCs represent a mixture of M-MDSCs (about 50%) and PMN-MDSCs (40%) (**Figure 1C**). To further define differences between both MDSC subpopulations, we analyzed expression of surface markers often coexpressed on CD11c-positive cells. CD11b+CD11c+ MDSCs exhibited increased expression of antigen-presenting cell (APC)-associated markers MHC class II (I-A^b^), F4/80, CD40, the activating costimulatory molecules CD80 and CD86, as well as the inhibitory molecules PD-L1 (CD274) and PD-L2 (CD273) (**Figure 1D**). These results clearly show that by using GM-CSF for MDSC generation *in vitro*, most of the cells exhibit the classical CD11b+CD11c− phenotype, while about 20% of cells showed coexpression of CD11c and APC-associated markers.

### CD11b+CD11c+ MDSCs Exhibit Increased Immunosuppressive Capacity Compared With CD11b+CD11c− MDSCs *In Vitro* and Shift the T-Cell Response Towards Type 2 Immunity

**Figure 2:**
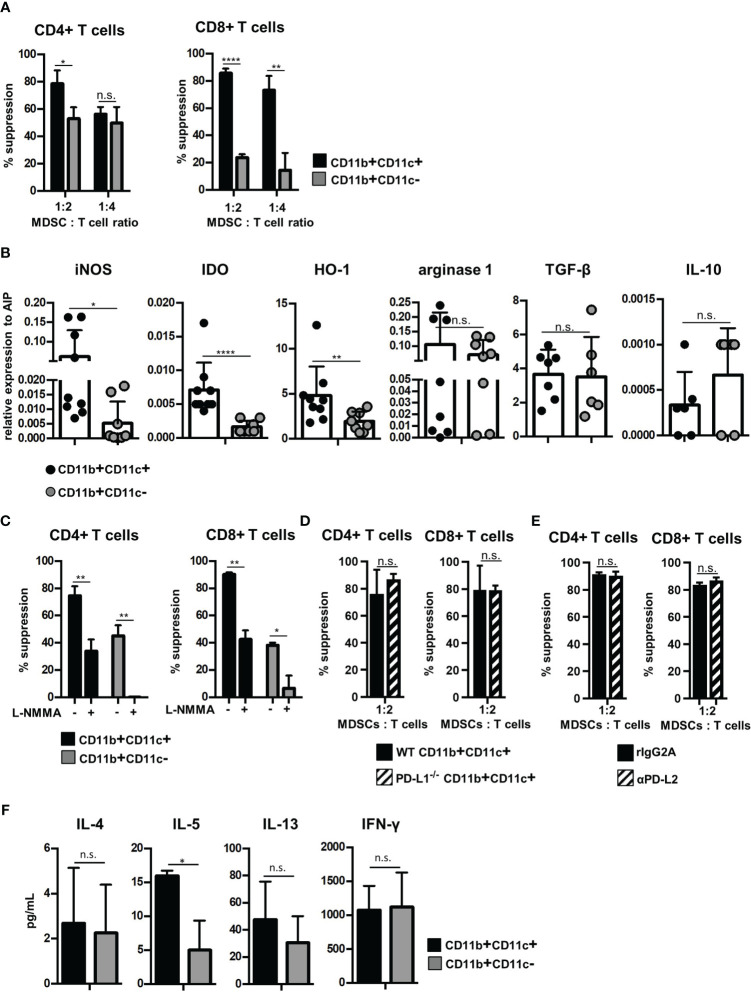
CD11b+CD11c+ MDSCs exhibit increased immunosuppressive capacity than CD11b+CD11c− MDSCs and induce type 2 immunity *in vitro*. CD11b+CD11c+ and CD11b+CD11c− MDSCs were isolated from B6-derived (H-2^b^, CD45.2+) *in vitro*-generated MDSCs. **(A)** CD11b+CD11c+ or CD11b+CD11c− MDSCs were cocultivated with B6.SJL-derived (H-2^b^, CD45.1+) CFSE-labeled spleen cells stimulated by irradiated allogeneic DBA/2-derived (H-2^d^, CD45.2+) spleen cells. After 4 days, CD45.1+ T cells were stained for CD3, CD4, and CD8 and suppression of CD4+ and CD8+ T-cell proliferation was calculated. **(B)** CD11b+CD11c+ and CD11b+CD11c− MDSC subpopulations were analyzed for relative expression of immunosuppressive molecules by qRT-PCRs. **(C)** CD11b+CD11c+ or CD11b+CD11c− MDSCs were cocultivated with B6.SJL-derived CFSE-labeled spleen cells stimulated by irradiated allogeneic DBA/2-derived spleen cells in the absence or presence of iNOS inhibitor L-NMMA (500 µM). After 4 days, suppression of CD4+ and CD8+ T-cell proliferation was determined. **(D)** B6-derived wildtype (WT) and PD-L1^−/−^ CD11b+CD11c+ or CD11b+CD11c− MDSCs were cocultured with CFSE-labeled B6.SJL-derived spleen cells stimulated with irradiated allogeneic DBA/2 spleen cells. **(E)** To block PD-L2, antagonistic PD-L2 antibodies or recombinant isotype control were added to MLRs, in which CFSE-labeled B6.SJL-derived spleen cells were stimulated with irradiated allogeneic DBA/2 spleen cells in the presence of CD11b+CD11c+ MDSCs. **(F)** CD11b+CD11c+ or CD11b+CD11c− MDSCs were cocultivated with B6.SJL-derived CFSE-labeled spleen cells stimulated by irradiated allogeneic DBA/2-derived spleen cells. After 4 days, secretion of cytokines associated with type 2 T-cell immunity (IL-4, IL-5, and IL-13) or type 1 T-cell immunity (IFN-γ) were determined in the supernatants. **(A)** Data represent the mean value ± SD of triplicates of one representative experiment out of four experiments performed. **(B)** Data represent the mean value ± SD of six to nine samples. **(C)** Data represent the mean value ± SD of triplicates of one representative experiment out of three experiments performed. **(D)** Data represent the mean value ± SD of *n* = 3 PD-L1^−/−^ and WT mice. **(E)** One experiment out of two experiments performed. Values present the mean value ± SD of triplicates. **(F)** Data represent the mean value ± SD of *n* = 3 experiments. **(A, C–F)** Student’s *t*-test. **(B)** Mann-Whitney *U* test. ^*^ *p* ≤ 0.05; ^**^ *p* ≤ 0.01; ^****^ *p* ≤ 0.0001. n.s., not significant.

Since CD11c is expressed on APCs such as dendritic cells, macrophages, and a small subset of B cells, we next defined whether CD11b+CD11c+ MDSCs exhibit immune-activating or suppressing functions. Therefore, B6-derived *in vitro*-generated MDSCs were separated by CD11c Micro Beads into CD11b+CD11c+ and CD11b+CD11c− MDSCs (**Supplementary Figure S1A**) with a purity of about 95% for both populations (**Supplementary Figure 1B**). Purified MDSCs were added at different numbers to CFSE-labeled B6-SJL (H-2^b^, CD45.1+) spleen cells, which were activated by DBA/2-derived (H-2^d^, CD45.2+) irradiated spleen cells. Using the congenic marker CD45.1 expressed solely on CFSE-labeled effector cells, proliferation of CD45.1+CD4+ and CD45.1+CD8+ T cells was determined. Both MDSC subpopulations efficiently suppressed T-cell proliferation, but CD11b+CD11c+ MDSCs exhibited strongly increased inhibitory capacity especially towards CD8+ T-cell proliferation (**Figure 2A**). Due to the differences in the immunosuppressive capacity, expression of molecules attributed to mediate suppression was defined in the isolated MDSC subsets by qRT-PCR. iNOS, IDO, and HO-1 expression were increased in CD11b+CD11c+ MDSCs while expression of arginase-1 and the anti-inflammatory modulators TGF-β and IL-10 were similar in both MDSC subsets (**Figure 2B**). By using the iNOS inhibitor L-NMMA, the immunosuppressive capacity of CD11b+CD11c− MDSCs towards CD4+ and CD8+ T cells was abolished to nearly 100%. L-NMMA-treated CD11b+CD11c+ MDSCs, however, maintained about 40% of their inhibitory function towards both T-cell subsets (**Figure 2C**). IDO or HO-1 inhibitors, however, did not affect the inhibitory capacity of CD11b+CD11c+ MDSCs (data not shown), indicating that immunosuppression is mediated by iNOS activity and a not yet identified mechanism. Since PD-L1 and PD-L2 were strongly upregulated on CD11b+CD11c+ MDSCs (**Figure 1A**) and are known to inhibit T-cell activation by binding to PD-1, impact of PD-L1 and PD-L2 on the suppressive function of CD11b+CD11c+ MDSCs was defined. PD-L1 function was abrogated by using isolated CD11b+CD11c+ MDSCs generated from BM cells of PD-L1^−/−^ mice and PD-L2 blocking was achieved by antagonistic antibodies. Purified PD-L1^−/−^ CD11b+CD11c+ MDSC added to allogeneic-activated spleen cells suppressed T-cell proliferation of CD4+ and CD8+ T cells comparable with PD-L1 expressing MDSCs derived from B6 wildtype (WT) mice (**Figure 2D**). Likewise, adding PD-L2 antagonistic antibodies to allogeneic-activated T cells in the presence of B6-derived CD11b+CD11c+ MDSCs did not impair T-cell suppression (**Figure 2E**) clearly showing that neither PD-L1 or PD-L2 contribute to CD11b+CD11c+ MDSC-mediated immunosuppression *in vitro*.

Although inhibition of T-cell expansion designates the main feature of MDSCs, MDSCs functions are also attributed to modulate the Th1/Th2 induction especially *in vivo*. To define the T-cell polarizing capacity of MDSC subsets *in vitro*, supernatants of allogeneic MLRs performed in the presence of CD11b+CD11c+ or CD11b+CD11c− were analyzed for composition of type 1- and type 2-asssociated cytokines. Of the type 2-specific cytokines analyzed, IL-5 secretion was strongly upregulated by CD11b+CD11c+ MDSCs, while IL-4 and IL-13 was unaffected. Th1-specific IFN-γ production was similar in CD11b+CD11c+- and CD11b+CD11c−-treated cultures (**Figure 2F**). In summary, these results show that CD11b+CD11c+ and CD11b+CD11c− MDSCs can be distinguished phenotypically and functionally.

### Exclusively the CD11b+CD11c+ MDSC Subset Prevents GVHD While Maintain the GVT Effect

**Figure 3:**
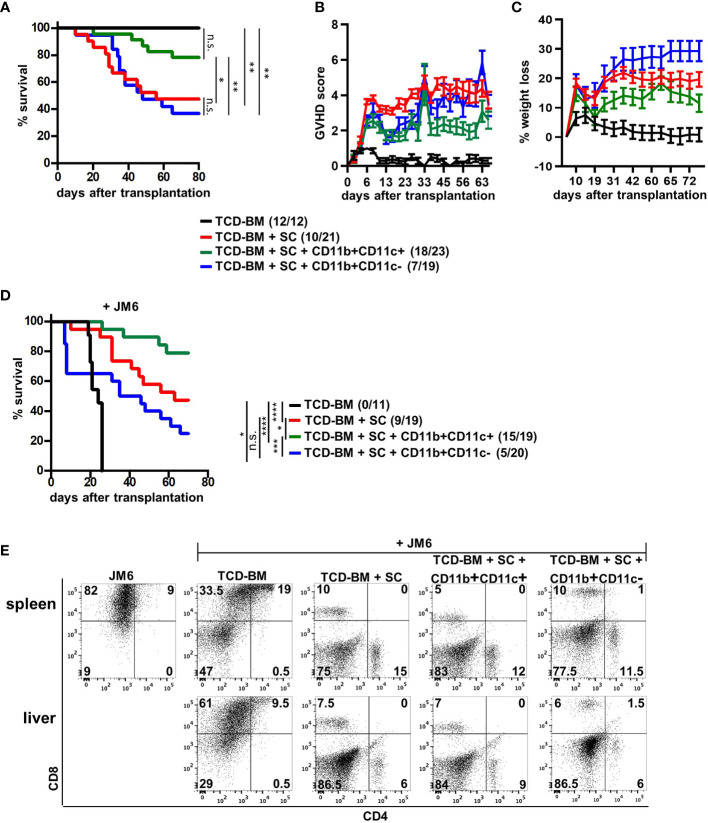
CD11b+CD11c+ expression defines the subpopulation of MDSCs able to prevent GVHD without disabling the GVT effect. **(A–E)** Lethally irradiated B6.bm1 mice (H-2K^bm1^) were reconstituted with B6-derived (H-2K^b^) TCD-BM and SCs with or without B6-derived CD11b+CD11c+ or CD11b+CD11c− MDSCs. **(D, E)** Mice were additionally coinjected with the CD8+CD4− syngeneic thymoma tumor cell line JM6 at day of transplantation. **(A, D)** Survival was determined. Surviving animals/total animals treated are indicated in brackets. **(B)** Clinical GVHD scores **(C)** and percentage of weight loss were determined. **(E)** Presence of tumor cells was analyzed in spleens and livers by staining for CD4 and CD8 at day mice were sacrificed due to their moribund state or at the end of the experiment. **(A, C)** Kaplan-Meier method and Log-rank test. **(B, C)** Data represent the mean value ± SEM. **(D)** Representative FACS diagrams of one mouse/group are displayed. ^*^ *p* ≤ 0.05; ^**^ *p* ≤ 0.01; ^***^ *p* ≤ 0.001; ^****^ *p* ≤ 0.0001; n.s., not significant.

Due to functional differences between both MDSC subsets *in vitro*, we tested their potential to block GVHD development after allogeneic BMT. We used the single MHC class I-disparate allogeneic BMT model, B6 (H-2K^b^)→B6.bm1 (H-K^bm1^), in which lethally irradiated B6.bm1 mice were reconstituted with TCD-BM and SCs from B6 mice. At the day of BMT, isolated CD11b+CD11c+ or CD11b+CD11c− MDSCs were cotransplanted together with TCD-BM and SCs. While 52% of mice transplanted with TCD-BM and SCs succumbed to the disease associated with high GVHD scores and weight loss of about 20%, CD11b+CD11c+ co-transplantation rescued 78% of the mice from disease-induced mortality reflected by a reduced GVHD score and less weight loss. In contrast, cotransplantation of CD11b+CD11c− MDSCs totally failed to prevent GVHD development. Surviving rates and GVHD scores were undistinguishable in mice receiving TCD-BM and SC and mice cotreated with CD11b+CD11c− MDSCs. Control mice receiving TCD-BM survived and did not develop GVHD (**Figures 3A–C**).

Maintenance of the GVT effect is a basic requirement for the application of allogeneic stem cell transplantation in the treatment of hematological malignancies. Therefore, the impact of MDSC subpopulations on the GVT effect was determined by coinjecting the CD8+CD4− syngeneic thymoma cell line JM6 in BM-reconstituted mice. All mice receiving only BM cells and JM6 died between 20 and 24 days after BMT from tumor development (**Figure 3D**) reflected by high numbers of tumor cells in spleen and liver (**Figure 3E**). Although transplantation of TCD-BM and SC totally prevented tumor growth in all mice due to the presence of tumor-reactive splenic mature T cells, 50% of the mice died by GVHD development. Most importantly, about 80% of the mice cotreated with CD11b+CD11c+ MDSCs survived reflected by the absence of tumor cells in spleen and liver. Five mice from this group died during the experiment. They were all tumor free but succumbed GVHD-induced death. Although all mice transplanted with CD11b+CD11c− MDSCs did not develop spleen or liver tumors, only 50% of the mice survived due to GVHD development, as shown in **Figure 3A**. Thus, our experiments define CD11b+CD11c+ MDSCs as the subpopulation of *in vitro*-generated MDSCs able to protect BMT mice from GVHD development without impairing antitumor cytotoxicity.

### CD11b+CD11c+-Mediated GVHD Inhibition Does Not Prevent Expansion and Homing of Allogeneic T Cells *In Vivo* But Requires Induction of Type 2 Immunity

**Figure 4:**
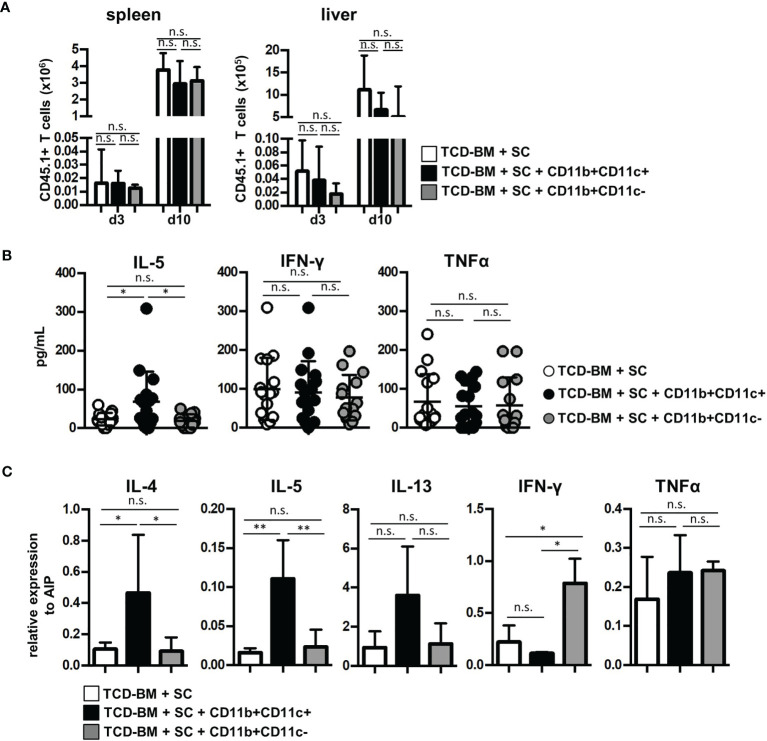
Cotransplantation of CD11b+CD11c+ MDSCs does not prevent allogeneic T-cell expansion and homing but induces type 2 immunity. **(A–C)** Lethally irradiated B6.bm1 (H-2K^bm1^, CD45.2) mice were reconstituted with B6-derived (H-2K^b^, CD45.2) TCD-BM and B6.SJL-derived (H-2K^b^, CD45.1) spleen cells in the presence or absence of B6-derived (H-2K^b^; CD45.2) CD11b+CD11c+ or CD11b+CD11c− MDSCs. **(A)** Spleen and liver were analyzed for infiltrated allogeneic CD45.1+ T cells 3 and 10 days after transplantation. **(B)** Ten days after transplantation, serum cytokine concentrations of Th2- (IL-5) and Th1-associated (IFN-γ and TNF-α) cytokines were determined. **(C)** Ten days after transplantation, splenic T cells were isolated and relative mRNA expression of Th2- (IL-4, IL-5, IL-13) and Th1-associated (TNF-α, IFN-γ) cytokines was analyzed by qRT-PCRs. **(A)** Data represent the mean value ± SD of three mice/group. Kruskal-Wallis test. **(B)** Data represent the mean value ± SD of 14–16 mice/group. **(C)** Data represent the mean value ± SD of *n* = 3–5 samples with cells from three to five pooled mice/sample. **(B, C)** ANOVA Tukey multiple comparison test. ^*^ *p* ≤ 0.05; ^**^ *p* ≤ 0.01. n.s., not significant.

Next, we questioned whether GVHD prevention by CD11b+CD11c+ MDSCs was due to impaired expansion of allogenic GVHD-inducing T cells, since CD11b+CD11c+ MDSCs most efficiently blocked allogeneic T-cell proliferation *in vitro*. By transplanting SCs from B6.SJL (CD45.1+) mice together with B6-derived TCD-BM (CD45.2+) into irradiated B6.bm1 (CD45.2+) mice, homing and expansion of allogeneic GVHD-inducing T cells were followed by staining the congenic marker CD45.1 in spleen and liver of transplanted mice. CD45.1+ T cells were detectable in spleen and the GVHD target organ liver already at day 3 after BMT in mice transplanted with TCD-BM and SCs. An increase of about 200-fold was achieved 10 days after BMT in both organs. However, cotransplantation of MDSCs did not prevent invasion and expansion of allogeneic T cells independent whether isolated CD11b+CD11c+ or CD11b+CD11c− MDSCs were transferred (**Figure 4A**). Ten days after BMT, allogeneic T-cell numbers continuously decreased and mice became lymphopenic at the time when clinical signs of GVHD were manifested (data not shown).

Since CD11b+CD11c+-mediated inhibition of GVHD did not impair allogeneic T-cell expansion, we determined whether CD11b+CD11c+ MDSCs support T-cell polarization towards Th2 immunity known to be advantageous for GVHD inhibition. Serum level of Th2-specific cytokine IL-5 was only elevated in mice treated with CD11b+CD11c+ MDSC. Type 2-specific cytokines such as IL-4 and IL-13 were not detectable, probably due to concentrations below the detection level of the kit used or degradation after freezing and thawing (41). Serum levels of Th1-associated cytokines IFN-γ or TNF-α were not altered by MDSC treatment (**Figure 4B**). To further prove CD11b+CD11c+-mediated type 2 polarization, mRNA expression of allogeneic T cells isolated from mice reconstituted with TCD-BM and SCs or cotransplanted with either CD11b+CD11c+ or CD11b+CD11c− MDSCs was determined. T cells isolated from CD11b+CD11c+ MDSC-treated mice expressed significantly increased levels of IL-4 and IL-5, while IL-13 was only slightly upregulated (**Figure 4C**). While TNF-α expression was unaffected by MDSC treatment, IFN-γ levels increased in T cells from CD11b+CD11c-treated mice further indicating that CD11b+CD11c− MDSCs support Th1 immunity and GVHD induction.

**Figure 5:**
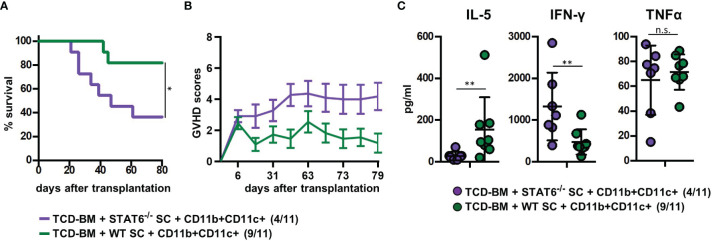
Type 2 immune induction by cotransplanted CD11b+CD11c+ MDSCs is required for GVHD prevention. Lethally irradiated B6.bm1 (H-2K^bm1^) recipient mice were reconstituted with B6-dervied (H-2K^b^) T-cell-depleted bone marrow (TCD-BM) and SCs either derived from B6 wild-type (WT) mice (H-2K^b^) or STAT6^−/−^ mice (H-2K^b^). B6-derived (H-2K^b^) CD11b+CD11c+ MDSCs were cotransplanted at day of transplantation. **(A)** Survival and **(B)** GVHD scores were analyzed. Surviving animals/total animals treated are indicated in brackets. **(C)** Ten days after transplantation, serum cytokine concentrations of Th2- (IL-5) and Th1-associated (IFN-γ and TNF-α) cytokines were determined. **(A)** Data represent the mean value ± SEM. **(B)** Kaplan-Meier method and Log-rank test. **(C)** Mann-Whitney *U* test. ^*^ *p* ≤ 0.05; ^**^ *p* ≤ 0.01; n.s., not significant.

To prove the indispensability of type 2 polarization for GVHD prevention in mice treated with CD11b+CD11c+ MDSCs, we reconstituted B6.bm1 mice with TCD-BM and allogeneic SCs either derived from STAT6-deficient (STAT6^−/−^) or B6 WT mice and CD11b+CD11c+ MDSCs. STAT6^−/−^ splenic T cells are unable to differentiate into type 2 T cells but retain their ability to turn into type 1 T cells and to induce GVHD indistinguishable from STAT6-expressing WT T cells as shown previously (18). Cotransplantation of CD11b+CD11c+ MDSCs in mice reconstituted with STAT6^−/−^ SCs failed to prevent GVHD and 64% of the mice succumbed to the disease, while only 18% of the mice receiving B6-derived WT SCs and CD11b+CD11c+ MDSCs developed lethal GVHD associated with increased GVHD scores (**Figures 5A, B**). In accordance to the survival data, IL-5 levels were only increased in mice reconstituted with B6-derived WT SCs, while IFN-γ levels were elevated in serum from mice reconstituted with STAT6^−/−^ SCs. Differences in the concentration of TNF-α levels were not detected (**Figure 5C**). In summary, these results clearly show that CD11b+CD11c+ MDSCs do not impair the expansion and homing of allogeneic T cells in lymphatic and GVHD target organs but prevent GVHD induction by shifting the T-cell response towards type 2 immunity.

### Comparative Transcriptome Analysis Between CD11b+CD11c+ and CD11b+CD11c− MDSCs

Since CD11b+CD11c+ and CD11b+CD11c− MDSCs can be clearly distinguished by their ability to interfere with GVHD development, we aimed to define genes and signaling pathways mediating immunosuppressive functions of CD11b+CD11c+ MDSCs in the context of BMT. Comparison of the transcriptome between CD11b+CD11c+ and CD11b+CD11c− MDSCs was done by mRNA-Seq. Principal component analysis (PCA) of two experiments displays the degree to which the transcriptome of CD11b+CD11c+ MDSCs differs from CD11b+CD11c− MDSCs. In both experiments, PCA analysis clearly separated two clusters corresponding to the CD11b+CD11c+ and CD11b+CD11c− MDSCs (**Supplementary Figure S2**), revealing a totally different transcriptome of both MDSC subpopulations which further underlies the functional differences between both subpopulations. In total, 2,783 differentially expressed genes (*p*-value <0.01, RPKM >5) were identified, from which 1,443 genes were upregulated and 1,340 genes were downregulated in CD11b+CD11c+ MDSCs compared with CD11b+CD11c− MDSCs. Focusing on the transcripts that were highly upregulated or downregulated in the CD11b+CD11c+ MDSC subpopulation, we performed enrichment analysis using GO database. Target genes upregulated in CD11b+CD11c+ MDSCs can be largely grouped into the biological and functional categories (**Table 1**): 1. cell movement and migration, 2. cell adhesion, 3. leukocyte activation and immune response, 4. ERK1 and ERK2 cascade, 5. response to cytokine, and 6. Stress response. Identified GO terms and linked target genes are listed in **Table 1** and **Supplementary Table S4**. Target genes downregulated in CD11b+CD11c+ MDSCs are mostly related to immune and defense response against other organisms such as bacteria or fungi, which might be related to their immunosuppressive phenotype. Identified GO terms and linked target genes are listed in **Table 2** and **Supplementary Table S5**. Focusing on the transcripts that were highly upregulated in CD11b+CD11c+ MDSCs, we ascertained the target genes that were upregulated more than 15-fold in CD11b+CD11c+ compared with CD11b+CD11c− MDSCs (**Table 3**). With a fold change of 57.96, CCL17 was the highest expressed target gene in CD11b+CD11c+ compared with CD11b+CD11c− MDSCs. Together with CCL22, which showed a 24-fold overexpression in CD11b+CD11c+ MDSCs, both chemokines are known to attract CCR4-bearing Th2 cells and serve as markers for the severity of Th2-mediated atopic dermatitis (42, 43). Furthermore, the fatty acid translocase CD36 is 20-fold stronger expressed in CD11b+CD11c+ MDSCs than in CD11b+CD11c− MDSCs and serves in association with the platelet-activating factor receptor as an important mediator of Th2-mediated house dust mite allergy development (44). Increased expression of CCL17, CCl22, and CD36 by CD11b+CD11c+ MDSC in comparison with CD11b+CD11c− MDSCs was confirmed by qRT-PCR. Additionally, CD36 was found to be strongly expressed on the surface of CD11b+CD11c+ MDSCs (**Supplementary Figure S3**). In summary, transcriptome analysis further underlines the functional differences between CD11b+CD11c+ and CD11b+CD11c− MDSCs and indicates candidate genes and pathways, which might contribute to the therapeutic potential of CD11b+CD11c+ MDSCs.

**Table 1:** GO term analysis and identification of biological and functional processes activated in CD11b+CD11c+ MDSCs compared with CD11b+CD11c− MDSCs.

Biological and functional category	GO term	GO ID	*p*-value changed	FDR *q*-value	Number of target genes
Cell movement and migration	Cell motility	0048870	2.50E−11	1.22E−07	62
	Cell migration	0016477	4.54E−11	1.47E−07	59
	Locomotion	0040011	5.08E−11	1.23E−07	65
	Lymphocyte migration	0072676	1.51E−10	2.44E−07	12
	Movement of cell or subcellular component	0006928	2.87E−10	3.98E−07	70
	Lymphocyte chemotaxis	0048247	3.36E−09	3.63E−06	8
	Chemotaxis	0006935	1.46E−08	1.18E−05	28
	Taxis	0042330	3.04E−08	1.85E−05	28
	Cell chemotaxis	0060326	9.10E−08	4.92E−05	22
	Monocyte chemotaxis	0002548	1.23E−07	5.97E−05	8
	Leukocyte chemotaxis	0030595	1.61E−07	6.80E−05	12
	Leukocyte migration	0050900	2.36E−07	8.49E−05	21
	Mononuclear cell migration	0071674	8.46E−07	2.00E−04	9
	Myeloid leukocyte migration	0097529	3.72E−06	6.57E−04	10
Cell adhesion	Biological adhesion	0022610	2.35E−08	1.53E−05	45
	Cell adhesion	0007155	8.20E−08	4.69E−05	43
	Positive regulation of cell-cell adhesion	0022409	1.22E−07	6.26E−05	16
	Positive regulation of cell adhesion	0045785	9.26E−07	2.14E−04	19
	Regulation of leukocyte cell-cell adhesion	1903037	3.40E−06	6.62E−04	15
	Regulation of cell-cell adhesion	0022407	4.05E−06	7.02E−04	39
Leukocyte activation and immune response	Immune response	0006955	1.47E−10	2.85E−07	55
	lymphocyte migration	0072676	1.51E−10	2.44E−07	12
	Positive regulation of immune system process	0002684	1.25E−09	1.52E−06	60
	Lymphocyte chemotaxis	0048247	3.36E−09	3.63E−06	8
	Monocyte chemotaxis	0002548	1.23E−07	5.97E−05	8
	Leukocyte chemotaxis	0030595	1.61E−07	6.80E−05	12
	positive regulation of lymphocyte activation	0051251	1.77E−07	7.17E−05	31
	Positive regulation of leukocyte activation	0002696	1.84E−07	7.14E−05	34
	Leukocyte migration	0050900	2.36E−07	8.49E−05	21
	Defense response	0006952	2.79E−07	9.69E−05	55
	Antigen processing and presentation of exogenous peptide antigen *via* MHC class II	0019886	5.15E−07	1.43E−04	9
	Antigen processing and presentation of peptide antigen *via* MHC class II	0002495	5.15E−07	1.47E−04	9
	Antigen processing and presentation of peptide or polysaccharide antigen *via* MHC class II	0002504	5.15E−07	1.52E−04	9
	Positive regulation of leukocyte cell-cell adhesion	1903039	5.40E−07	1.46E−04	14
	Negative regulation of immune system process	0002683	7.14E−07	1.78E−04	21
	Positive regulation of T-cell activation	0050870	8.39E−07	2.04E−04	13
	Mononuclear cell migration	0071674	8.46E−07	2.00E−04	9
	Adaptive immune response	0002250	1.40E−06	3.17E−04	12
	Humoral immune response	0006959	3.25E−06	6.45E−04	15
	Regulation of leukocyte cell-cell adhesion	1903037	3.40E−06	6.62E−04	15
	Positive regulation of leukocyte differentiation	1902107	3.58E−06	6.82E−04	23
	Myeloid leukocyte migration	0097529	3.72E−06	6.57E−04	10
	Inflammatory response	0006954	4.71E−06	7.89E−04	35
	Regulation of leukocyte differentiation	1902105	5.18E−06	8.53E−04	12
	Antigen processing and presentation of exogenous peptide antigen	0002478	6.45E−06	1.03E−03	9
	Myeloid leukocyte migration	0097529	3.72E−06	6.57E−04	10
ERK1 and ERK2 cascade	Regulation of ERK1 and ERK2 cascade	0070372	3.59E−06	6.71E−04	16
	Positive regulation of ERK1 and ERK2 cascade	0070374	2.31E−08	1.60E−05	16
Response to cytokine	Chemokine-mediated signaling pathway	0070098	8.52E−09	7.53E−06	11
	Response to interferon-gamma	0034341	2.09E−08	1.57E−05	20
Response to stress	Response to tumor necrosis factor	0034612	1.94E−06	4.29E−04	9

**Table 2:** GO term analysis and identification of biological and functional processes downregulated in CD11b+CD11c+ MDSCs compared with CD11b+CD11c− MDSCs.

Biological and functional category	GO term	GO ID	*p*-value changed	FDR *q*-value	Number of target genes
Immune and defense response	Defense response	0006952	1.41E−09	1.37E−05	20
	Defense response to fungus	0050832	1.31E−07	6.37E−04	5
	Defense response to bacterium	0042742	3.49E−07	8.48E−04	9
	Antifungal humoral response	0019732	5.40E−07	1.05E−03	2
	Response to bacterium	0009617	1.13E−06	1.83E−03	11
	Response to fungus	0009620	1.57E−06	2.18E−03	5
	Disruption of cells of other organism	0044364	1.94E−06	2.36E−03	6
	Killing of cells of other organism	0031640	1.94E−06	2.10E−03	6
	Regulation of inflammatory response	0050727	2.09E−06	2.03E−03	28
	Organ- or tissue-specific immune response	0002251	2.41E−06	1.95E−03	5
	Mucosal immune response	0002385	2.41E−06	2.13E−03	5
	Innate immune response in mucosa	0002227	2.45E−06	1.83E−03	3
	Regulation of defense response	0031347	2.53E−06	1.75E−03	42
	Humoral immune response	0006959	4.08E−06	2.64E−03	7

**Table 3:** Most strongly upregulated genes (>15-fold increase) in CD11b+CD11c+ MDSCs compared with CD11b+CD11c− MDSCs.

	Target gene	Name	Fold change	Adjusted *p*-value
1.	CCL17	Chemokine (C–C motif) ligand 17	57.96	9.55E−08
2.	Plet1	Placenta-expressed transcript 1 protein	43.11	2.33E−07
3.	Hepacam2	HEPACAM family member 2	42.23	4.02E−05
4.	Klrb1b	Killer cell lectin-like receptor subfamily B member 1B allele B	36.63	5.39E−06
5.	Hr	Lysine-specific demethylase hairless	34.35	8.50E−08
6.	H2-Eb1	H-2 class II histocompatibility antigen, I-A beta chain	32.28	9.07E−07
7.	Emp2	Epithelial membrane protein 2	30.40	3.74E−04
8.	C1qc	Complement C1q subcomponent subunit C	28.67	5.17E−03
9.	H2-Aa	H-2 class II histocompatibility antigen, A-B alpha chain	28.65	5.25E−07
10	Kcp	Kielin/chordin-like protein	27.77	3.52E−03
11.	Flnc	Filamin-C	26.75	2.97E−06
12.	Aldh1a2	Retinal dehydrogenase 2	26.16	3.67E−04
13.	H2-Ab1	H-2 class II histocompatibility antigen, A beta chain	25.73	2.51E−07
14.	Adam23	Disintegrin and metalloproteinase domain-containing protein 23	25.64	3.40E−05
15.	Speg	Striated muscle-specific serine/threonine-protein kinase	25.16	9.10E−06
16.	C1qb	Complement C1q subcomponent subunit B	24.72	2.47E−03
17.	CCL22	Chemokine (C–C motif) ligand 22	24.45	8.61E−05
18.	Mmp12	Macrophage metalloelastase 12	24.12	3.21E−06
19.	Sema6d	Semaphorin-6D	24.00	1.74E−08
20.	Tnfaip8l3	Tumor necrosis factor alpha-induced protein 8-like protein 3	23.94	6.67E−07
21.	Itgae	Integrin alpha-E	23.02	6.81E−03
22	Dcstamp	Dendritic cell-specific transmembrane protein	22.63	1.83E−06
23.	Nr4a3	Nuclear receptor subfamily 4 group A member 3	22.28	5.09E−04
24.	Fscn1	Fascin	22.01	1.21E−03
25.	Ciita	MHC class II transactivator	21.69	8.50E−08
26.	CCR7	C–C chemokine receptor type 7	21.55	4.26E−03
27	Tnfrsf9	Tumor necrosis factor receptor superfamily member 9	21.35	3.11E−06
28	Asgr2	Asialoglycoprotein receptor 2	21.24	2.30E−03
29.	Anpep	Aminopeptidase N	21.00	1.94E−05
30.	Hgfac	Hepatocyte growth factor activator	20.47	1.59E−04
31.	Ptx3	Pentraxin-related protein PTX3	20.36	1.60E−04
32.	CD36	Platelet glycoprotein 4	19.65	1.10E−07
33.	IL7r	Interleukin-7 receptor subunit alpha	19,36	9.62E−04
34.	P2rx5	Purinergic receptor P2X ligand-gated ion channel 5	18.08	3.51E−08
35.	Tspan33	Tetraspanin-33	17.87	2.09E−05
36.	Blnk	B-cell linker	17.66	8.97E−05
37.	Il4i1	Interleukin 4 induced 1	16.61	2.46E−04
38.	Zbtb46	Zinc finger and BTB domain containing 46	16.35	4.66E−06
39.	Sdc3	Syndecan 3	15.04	2.41E−07

## Discussion

Allogeneic hematopoietic cell transplantation is considered an important treatment strategy to cure life-threatening malignant hematological diseases, however, with the limitation of GVHD development. Initial treatment comprises steroid therapy, while second-line treatment often includes immunomodulatory therapies to dampen the destructive capacity of allogeneic T cells. MDSCs are recognized as strong modulators of T-cell functions and were already applied in preclinical models as cellular therapy for GVHD prevention. Considering the heterogenicity of *in vitro*-generated MDSCs, we aimed to define the MDSC subset responsible for GVHD prevention. To our knowledge, we show here for the first time that only a small proportion of MDSCs, which have been generated *in vitro* form BM cells, fulfills GVHD-inhibiting functions. This subset is characterized by the coexpression of Gr-1, CD11b, and CD11c. Gr-1+CD11b+CD11c+ MDSCs effectively prevent GVHD development and maintain antitumor cytotoxicity of allogeneic T cells, while the majority of the *in vitro*-generated MDSCs expressing Gr-1+CD11b+CD11c− are totally inefficient to dampen GVHD, although they block T-cell expansion *in vitro*. Extensive differences in the transcriptomic landscape of both populations underlined their various *in vivo* functions, indicating that the success of cellular therapies using MDSCs requires a thoughtful characterization of MDSC subset functions *in vitro* and *in vivo*.

In a clinically relevant BMT model with disparity in only one MHC molecule (B6 into B6.bm1), we defined which subset of *in vitro*-generated MDSCs prevents GVHD. MDSC were generated from BM cells in the presence of GM-CSF. More than 90% of the cells exhibited Gr-1 and CD11b expression, but only a small proportion of about 10%–25% cells showed coexpression of the integrin alphaX CD11c, which is also found at high levels not only on the surface of dendritic cell, but also on monocytes, macrophages, neutrophils, and subsets of NK, B, and T cells. By separating MDSCs into CD11b+CD11c+ and CD11b+CD11c− subsets, a clear correlation with the classically defined M-MDSCs and PMN-MDSCs was not observed. While CD11b+CD11c− MDSCs represented a mixture of M-MDSCs and PMN-MDSCs, CD11b+CD11c+ MDSCs consisted mainly of M-MDSCs, a small proportion of PMN-MDSCs and cells, which neither expressed Ly-6C nor Ly-6G. CD11b+CD11c+ MDSCs further expressed higher levels of APC-associated markers such as CD80, CD86, MHC class II, and F4/80 compared with their CD11c-negative counterparts. Importantly, a single injection of CD11b+CD11c+ MDSCs inhibited GVHD development in about 80% of the BM-transplanted mice, while adoptive transfer of CD11b+CD11c− MDSCs had no impact on disease development. CD11b+CD11c+-treated mice, however, remain immunosufficient since syngeneic tumor cells were efficiently eradicated in 100% of the mice. Although BMTs are routinely applied to abrogate residual B-cell lymphoma cells, we used the CD8^+^CD4^−^ JM6 thymoma cell line. To our knowledge, JM6 is currently the only available syngeneic tumor cell line for B6.bm1 mice. By using JM6 cells, we cannot totally exclude that MDSCs interact with JM6 tumor growth in transplanted mice. However, in a previous work, unseparated MDSCs, which represent a mixture of CD11b+CD11c+ and CD11b+CD11c− MDSCs, did not abrogate the GVT effect in a parent into F1 BMT model (18), indicating that none of the MDSC subpopulations reduce the capacity of allogeneic T cells to attack residual tumor cells. Furthermore, the GVHD-inhibiting capacity of CD11b+CD11c+ MDSC requires confirmation in other BMT models with disparities also in only MHC class II genes or disparities in MHC class I and II genes to exclude that the observed effects are model dependent.

*In vitro* or *in vivo* induction of MDSCs for cellular therapy of GVHD have been performed by using various approaches with different effectiveness (45); however, the ability of different MDSC subpopulations have not been analyzed for their GVHD-inhibiting potential. Treatment of donor mice with CpG and incomplete Freund’s adjuvant (IFA), G-CSF, or recombinant G-CSF/Flt-3 ligand + G-CSF results in increase of splenic CD11b+Gr1+ cells preventing GVHD after cotransplantation with allogeneic T cells (14–16). While a proportion of CpG+IFA-induced Gr-1 cells coexpress CD80, CD86, and CD11c (15), CD11c expression is absent on *in vivo*-generated G-CSF-or G-CSF/Flt-3 + G-CSF-induced MDSCs (14, 16). MDSCs induced *in vitro* from BM cells by GM-CSF, G-CSF, and IL-13 exhibited expression of CD11c on about 15% of the cells, but adoptive transfer into BMT mice was performed solely with unseparated MDSCs (20). However, GM-CSF+G-CSF+IL-13-induced MDSCs upregulated CD11c, MHC class II, and F4/80 in the inflammatory GVHD environment. Re-isolation of CD11c+, MHC class II^high^, and F4/80^high^ cells from GVHD mice showed a loss in immunosuppressivity *in vitro* (46). Despite the expression of surface makers similar to *in vitro*-generated CD11b+CD11c+ MDSCs, functional properties are different. While *ex vivo*-isolated CD11c+ MDSCs mediate T-cell suppression by arginase-1, suppressive ability by *in vitro*-generated CD11b+CD11c+ MDSCs was mainly attributed to iNOS activity and a not yet defined mechanism, which does not involve IDO, arginase-1, or HO-I activity. Even the role of PMN-MDSCs and M-MDSCs for GVHD development is not defined since we are not aware of BMT experiments using isolated PMN- or M-MDSCs as suppressor cells. G-CSF treatment of donor mice induced low-density splenic granulocytes, which inhibit experimental GVHD (47) and the administration of GVHD-suppressing drug rapamycin, results in expansion of PMN-MDSCs (48) indicating that PMN-MDSCs are the major suppressor population. On the other hand, the presence of G-CSF-induced M-MDSCs correlates with a lower GVHD incidence in humans and humanized GVHD models (21, 49, 50). These findings might reflect species-specific differences in the dependence on MDSC subsets for GVHD inhibition, but extracorporeal photopheresis promotes protective PMN-MDSC expansion in GVHD patients (51).

Striking differences were observed in the *in vitro* and *in vivo* activity of MDSC subsets. While CD11b+CD11c− MDSCs suppressed allogeneic T-cell expansion *in vitro* although to a lesser extent than CD11b+CD11c+ MDSCs, they totally failed to prevent GVHD induction. Immunosuppressive mechanisms differ in both subpopulations since CD11b+CD11c− MDSCs inhibit T-cell proliferation *in vitro* exclusively by iNOS activity, while function of CD11b+CD11c+ MDSCs depends only half on iNOS. Despite upregulation of PD-L1 and PD-L2, both molecules are neglectable for immunosuppression *in vitro* by CD11b+CD11c+ MDSCs. The discrepancy of *in vitro* and *in vivo* action of MDSCs is supported by our work. Unseparated *in vitro*-generated MDSCs induced from BM cells by GM-CSF strongly suppressed T-cell proliferation *in vitro*, but act immunostimulatory in mice receiving blunt chest trauma (TxT). MDSC treatment of TxT mice strongly increased splenic T-cell numbers and proliferative capacity without impairing antigen reactivity (52). Studies by Schmidt et al. also show that tumor-induced MDSCs prevent cytotoxic T lymphocyte (CTL) functions *in vitro* but not *in vivo* following adoptive transfer (53) strongly indicating an important effect on MDSC functions by the interacting microenvironment. Likewise, MDSCs isolated from septic mice at different time points after sepsis induction and transferred into septic mice either deteriorate or ameliorate disease development (54).

Microenvironmental influence on MDSC function is further underlined by the finding that CD11b+CD11c+ MDSCs prevent GVHD development by inducing Th2 immunity without altering allogeneic T-cell expansion and homing, although T-cell expansion was severely blocked by this subpopulation *in vitro*. MDSC-mediated type 2 immunity induction is reported also in the context of cancer, sepsis, pregnancy, and virus infection (5, 6, 8, 55). On the other hand, the transfer of MDSCs in models of Th2-mediated diseases such as asthma-related airway inflammation dampens disease development by shifting immune responses towards Th1 immunity (9, 10). Interestingly, Th1 immunity induction by MDSCs in asthma-related models is found independent whether MDSCs were derived from LPS-treated or tumor-bearing mice, although MDSCs in the context of cancers are known to promote Th2 immunity. Inhibiting T-cell proliferation *in vitro* is indispensable for their assignment as MDSCs (26) but is not necessarily indicative for their *in vivo* functions.

Defining MDSC subsets either able to prevent GVHD or being totally inefficient in blocking GVHD development opens up the possibility to define molecules and molecular pathways contributing to MDSC-mediated GVHD inhibition. mRNA-Seq analysis showed that CD11b+CD11c+ and CD11b+CD11c− MDSCs had a totally different transcriptomic landscape differing in more than 2,500 genes. Upon the most strongly upregulated genes (>15-fold increase), the fatty acid translocase CD36 or the chemokines CCL17 and CCL22 were identified. Although CD36 expression is not directly linked to elevated immunosuppressivity, increased lipid contents are reported to augment the immunosuppressive functions of MDSCs (56–58), and Baumann et al. recently reported that human MDSCs derived from isolated CD14+ blood monocytes downregulate glycolysis-related enzymes (59). CCL17 and CCL22 are key chemokines inducing Th2 chemotaxis and are strongly elevated in the serum of patients with Th2-driven atopic dermatitis (42, 43). Possibly, Th2 cells are attracted into lymphatic areas invaded by CCL17/CCL22 expressing CD11b+CD11c+ MDSCs and stimulated for increased expansion. However, only adoptive transfer experiments with MDSCs derived from CD36 or CCL17/CCL22-deficient mice will clarify their substantial role in GVHD prevention. Due to the high numbers of differentially expressed genes, it might be worthwhile to re-isolate adoptively transferred CD11b+CD11c+ and CD11b+CD11c− MDSCs from BM-transplanted mice for transcriptome analysis. Defining the intersection of genes differentially expressed by *in vitro* and *ex vivo* isolated CD11b+CD11c+ MDSCs might narrow down the number of possible candidates responsible for GVHD prevention.

Taken together, we could define a small subset of GM-CSF-induced MDSCs characterized by the coexpression of Gr-1+CD11b+CD11c+ as the MDSC subpopulation able to prevent GVHD while maintaining T-cell reactivity and cytotoxicity. This might offer the possibility to identify key molecules and signaling pathways involved in disease prevention with the future perspective to substitute cellular MDSC therapy by pharmacological approaches. Furthermore, the clear discrepancy between *in vitro* and *in vivo* functions of MDSCs requires thoughtful testing of MDSC functions in the relevant disease context.

## Data Availability Statement

The original contributions presented in the study are publicly available and are based on JS's dissertation: (60). mRNA Seq data can be found here: https://www.ncbi.nlm.nih.gov/geo/query/acc.cgi?acc=GSE182262.

## Ethics Statement

The animal study was reviewed and approved by the ethical committee Regierungspräsidium Tübingen, Germany.

## Author Contributions

JS performed experiments, analyzed data, and generated figures. KK, HH, SP, and KF-C performed mRNA-Seq analysis and analyzed transcriptomic data. K-MD contributed to the study design and interpreted data. GS created the study design, performed data interpretation, and wrote the manuscript together with JS. All authors approved the final version of the manuscript, revised the manuscript, and are accountable for all respects of the work.

## Funding

This study was supported by Boehringer Ingelheim Ulm University BioCenter (TPI2) and the International Graduate School in Molecular Medicine, Ulm, Germany.

## Conflict of Interest

KK, HH, KF-C, and SP are employed by Boehringer Ingelheim Pharma Co. KG and Boehringer Ingelheim RCV GmbH & Co. KG.

The remaining authors declare that the research was conducted in the absence of any commercial or financial relationships that could be construed as a potential conflict of interest.

## Publisher’s Note

All claims expressed in this article are solely those of the authors and do not necessarily represent those of their affiliated organizations, or those of the publisher, the editors and the reviewers. Any product that may be evaluated in this article, or claim that may be made by its manufacturer, is not guaranteed or endorsed by the publisher.
