# Supplementary material for: Graft-Versus-Host Disease Prevention by In Vitro-Generated Myeloid-Derived Suppressor Cells Is Exclusively Mediated by the CD11b+CD11c+ MDSC Subpopulation
Source: Front Immunol. 2021 Oct 14;12:754316. doi: 10.3389/fimmu.2021.754316 (PMC8551363; doi:10.3389/fimmu.2021.754316)
Supplement: Supplementary file 1 [file DataSheet_1.pdf]

A

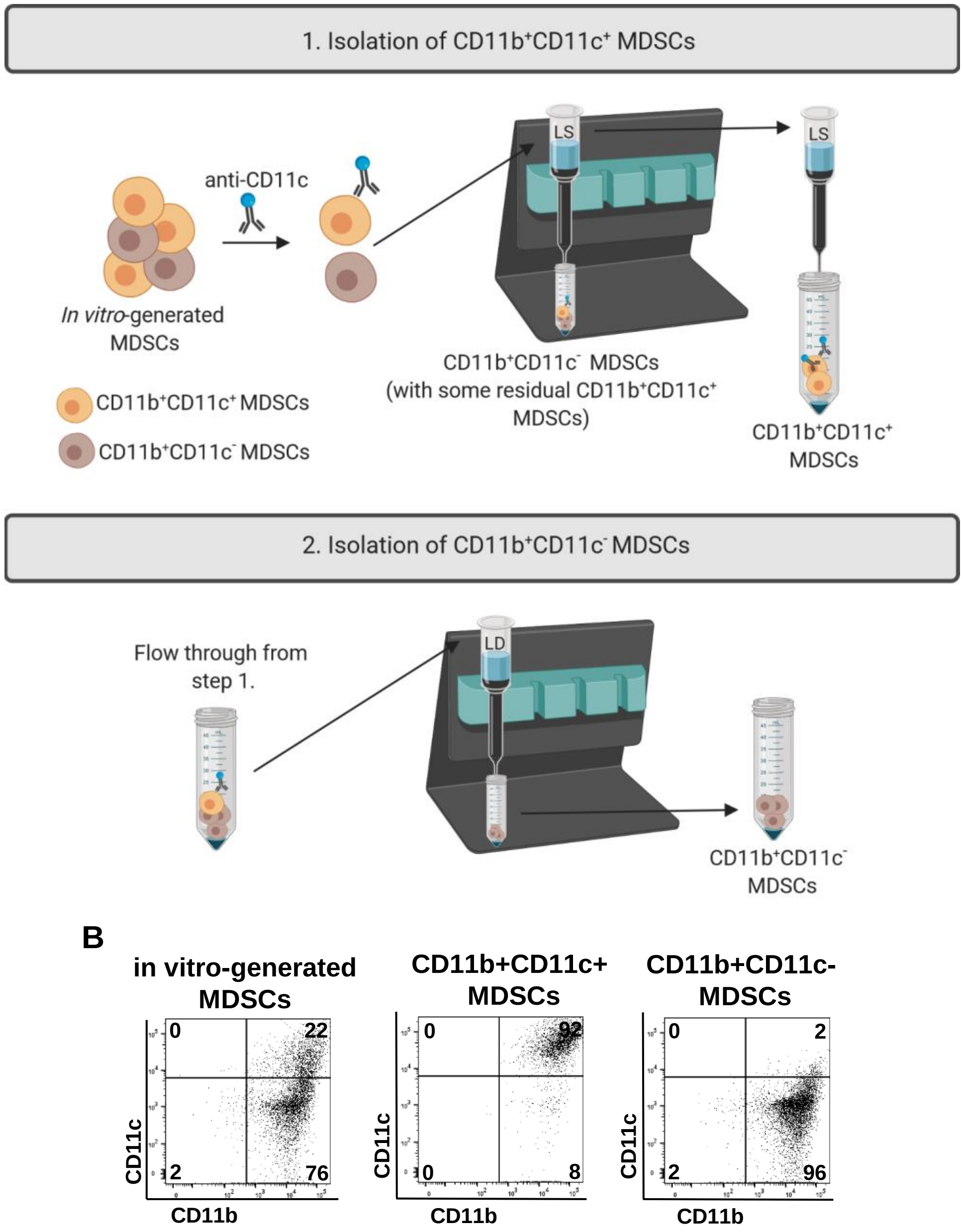

Suppl. Figure 1: Isolation of CD11b+CD11c+ and CD11b+CD11c- MDSCs. (A) MDSC subpopulations were isolated from in vitro-generated MDSCs by MACS technology. CD11b+CD11c+ MDSCs were isolated by anti-CD11c MicroBeads and performing a positive selection with LS columns. CD11b+CD11c- MDSCs were isolated from the flow through of the CD11b+CD11c+ isolation. To deplete remaining CD11b+CD11c+ cells from the flow through, flow through was loaded on depleting LD columns. **(B)** Data show a representative flow cytometry graph indicating purity of isolated CD11b+CD11c+ and CD11b+CD11c- MDSC subpopulations isolated from in vitro-generated MDSCs. **(A)** Figure created with Biorender.com. **(B)** FACS diagrams of one representative experiment are shown.

Suppl. Figure 1

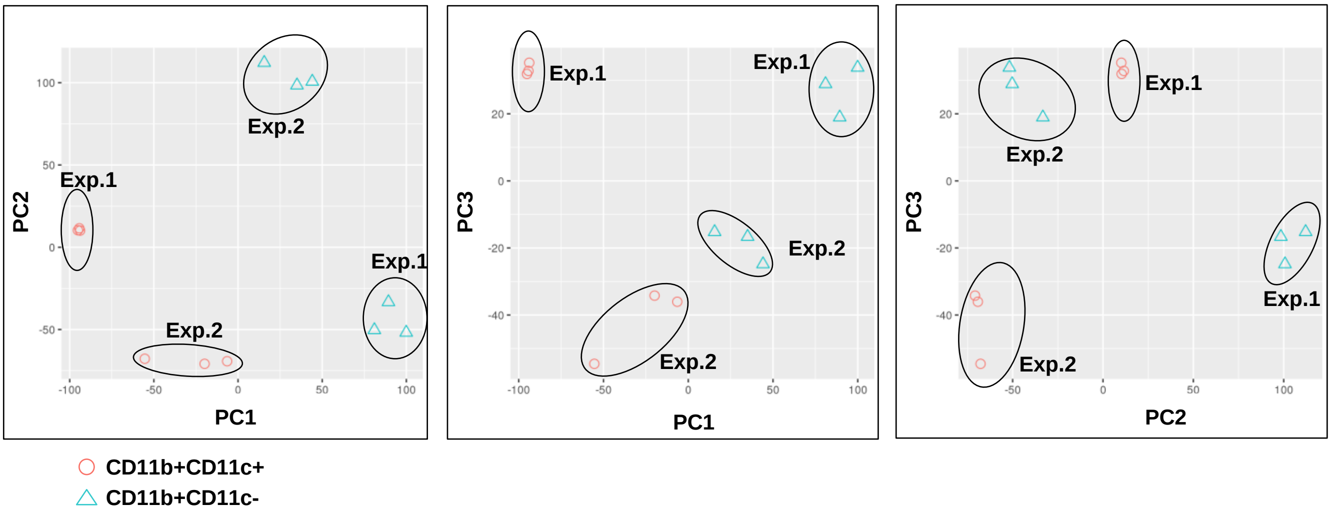

Suppl. Figure 2: Principal component analysis (PCA) recapitulates strong differences in the transcriptome between CD11b+CD11c+ and CD11b+CD11c- MDSCs. CD11b+CD11c+ and CD11b+CD11c- MDSCs were isolated from in vitro-generated MDSCs after 4 days in culture. Principal component analysis was performed with three samples/ group from two independent performed experiments. Each dot/triangle represents one array sample. In both experiments PCA analysis clearly separated two clusters corresponding to the CD11b+CD11c+ and CD11b+CD11c- MDSCs.

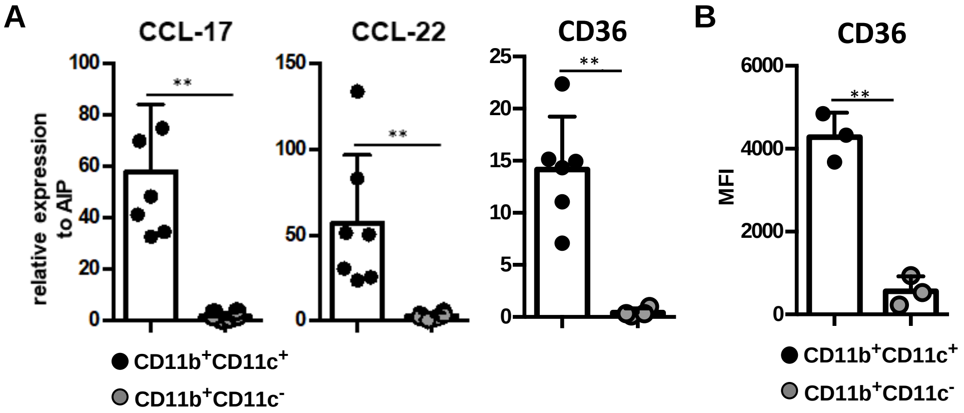

Suppl. Figure 3: CD11b+CD11c+ MDSCs express higher levels of CCL17, CCL22 and CD36 compared to CD11b+CD11c- MDSCs. (A) CD11b+CD11c+ and CD11b+CD11c- MDSC subpopulations were isolated from in vitro-generated MDSCs after 4 days in culture. mRNA expression of CCL-17, CCL-22 and CD36 was analysed by qRT-PCR and relative expression was calculated. **(B)** CD36 surface expression was analyzed by flow cytometry and mean fluorescence intensity (MFI) of CD36 was calculated. **(A)** Data represent the mean value ± SD of 4-7 samples/group. **(B)** Data represent the mean value ± SD of 3 samples/group. **(A-B)** Mann-Whitney test. **≤p0.01.

Suppl. Tab. S1: Mice

Mouse strain	Short form	Genotype	Sex	Age	Supplier
**B6-C-H2-K<bm1>/ByJ**	**B6.bm1**	H-2K^{bm1}, CD45.2	Female	9-20 weeks	Bred at University of Ulm; originally obtained from Jackson Laboratory
**B6.SJL-Ptprc^{a}Pep3^{b}/BoyJ**	**B6.SJL**	H-2^{b}; CD45.1	Female	6-9 weeks	Bred at University of Ulm; originally obtained from Jackson Laboratory
**B6.129(C)-Stat6^{tm1Gru}/J**	**STAT6$^{-/-}$**	H-2^{b}	Female	6-9 weeks	Bred at University of Ulm; originally obtained from Jackson Laboratory
**B7-H1 deficient mice** [1]	**PD-L1$^{-/-}$**	H-2^{b}	Female	6-9 weeks	Bone marrow cells provided by R. Schirmbeck; Internal Medicine I, University Medical Center Ulm
**C57BL/6**	**B6**	H-2^{b}; CD45.2	Female	6-9 weeks	Janvier
**DBA/2**		H-2^{d}; CD45.2	Female	6-9 weeks	Janvier

[1] Dong H, Zhu G, Tamada K, Flies DB, van Deursen JMA, Chen L (2004) B7-H1 Determines Accumulation and Deletion of Intrahepatic CD8+ T Lymphocytes. Immunity 20:327–336

Suppl. Tab. S2. Antibodies for flow cytometry

target protein	supplier	clone	fluorochrom	category	RRID
**CD11b**	ThermoFisher Scientfic	M1/70	APC<br>Alexa Fluor 700	56-0112-80<br>17-0112-81	AB_657586<br>AB_469342
**CD11c**	ThermoFisher Scientfic	N418	PE<br>Pe-Cyanine 7	12-0114-81<br>25-0114-81	AB_465551<br>AB_469589
**CD36**	BioLegend	HM36	PE	102605	AB_389348
**CD3ε**	ThermoFisher Scientfic	145-2C11	Pe-Cyanine 7	25-0031-81	AB_469571
**CD4**	ThermoFisher Scientfic	GK1.5	APC-eFluor 780	47-0041-80	AB_11219883
**CD40**	ThermoFisher Scientfic	1C10	APC	17-0401-81	AB_469385
**CD45.1**	BD Biosciences	A20	V450	560520	AB_1727490
**CD80**	ThermoFisher Scientfic	6-10A1	PE	12-0801-81	AB_465751
**CD86**	ThermoFisher Scientfic	GL1	FITC	11-0862-81	AB_465147
**CD8a**	ThermoFisher Scientfic	53-6.7	APC	17-0081-81	AB_469334
**F4/80**	ThermoFisher Scientfic	BM8	eFluor 450	48-4801-80	AB_1548756
**Gr-1**	ThermoFisher Scientfic	RB6-8C5	eFluor 450	48-5931-80	AB_1548797
**I-Ab**	BD Biosciences	AF6-120.1	FITC	553551	AB_394918
**Ly-6C**	ThermoFisher Scientfic	HK1.4	APC-eFluor 780	47-5932-80	AB_2573991
**Ly-6G**	BD Biosciences	1A8	V450	560603	AB_1727564
**PD-L1**	ThermoFisher Scientfic	MIH5	PE	12-5982-81	AB_466088
**PD-L2**	ThermoFisher Scientfic	122	FITC	11-9972-81	AB_465461

Suppl. Tab. S3. Primer for qRT-PCR

target	sequence (5´→3´)
**AIP forward**	GCTCCGTTATAGATGACAGC
**AIP reverse**	ATCTCGATGTGGAAGATGAG
**arginase 1 forward**	TCCTTTCAAATTGTGAAGAACCCACGGTC
**arginase 1 reverse**	AGAATCCTGGTACATCTGGGAACTTTCCT
**CCL17 forward**	ATCCCTGGAACACTCCACTG
**CCL17 reverse**	TGCTTCTGGGGACTTTTCTG
**CCL22 forward**	TCCTCCTCCCTAGGACAGTTT
**CCL22 reverse**	TCTGGACCTCAAAATCCTGC
**CD36 forward**	TCGGAACTGTGGGCTCATTGC
**CD36 reverse**	GCCACGTCATCTGGGTTTTGC
**HO-1 forward**	TCAAGGCCTCAGACAAATCC
**HO-1 reverse**	ACAACCAGTGAGTGGAGCCT
**IDO forward**	GGATGCCTGACTTTGTGGAC
**IDO reverse**	TTCTTTGCCAGCCTCGTGT
**IFN-γ forward**	TGCAGAGCCAGATTATCTCTTTCTACCTCAG
**IFN-γ reverse**	GGTTGTTGACCTCAAACTTGGCAATACTC
**IL-10 forward**	CACTGCTATGCTGCCTGCTCTTACTGAC
**IL-10 reverse**	TGGCAACCCAAGTAACCCTTAAAGTCCT
**IL-13 forward**	CACACTCCATACCATGCTGC
**IL-13 reverse**	TGTGTCTCTCCCTCTGACCC
**IL-4 forward**	GGTGTTCTTCGTTGCTGTGA
**IL-4 reverse**	TCTCGAATGTACCAGGAGCC
**IL-5 forward**	CCCACGGACAGTTTGATTCT
**IL-5 reverse**	GCAATGAGACGATGAGGCTT
**iNOS forward**	AGCAATGGGCAGACTCTGAAGAAATCTC
**iNOS reverse**	ATGTTTGCTTCGGACATCAAAGGTCTCAC
**TGF-β forward**	TCTACCAGAAATATAGCAACAATTCCT
**TGF-β reverse**	CTGAATCGAAAGCCCTGTATTCCGTCTC
**TNFα forward**	CCAGACCCTCACACTCAGATCA TCTTCTC
**TNFα reverse**	CTAGTTGGTTGTCTTTGAGATCCATGCCGT

Suppl. Tab. S4: GO terms of biological processes and linked target genes up-regulated in CD11b+CD11c+ MDSCs compared to CD11b+CD11c- MDSCs

**1.) cell movement and migration**		
**biological process**	**GO ID**	**linked target genes**
cell motility	0048870	Nfatc2, Cxcl3, Lamc1, Emp2, Eng, Fscn1, Jup, rab13, Mmp14, 1600029D21Rik, Nrp2, Nrp1, Axl, Mmp12, Slamf8, Cxcl16, Pdpn, Ccl17, Ccl22, Plekho1, Itgb7, Ext1, Ch25h, Rhoc, Acvr1, Cd2ap, Nav1, Acvrl1, Plekhg5, Tns1, Sema6d, Tubb2a, Nrg1, Gpr183, Cxcl2, Cttn, Ccl9, Flrt3, Gpr35, Vav2, C230081A13Rik, Cx3cl1, Tesk1, Megf8, Ccl7, Pmp22, Ccr7, Lgals3, Mertk, Ptpro, Amica1, Mcoln2, Insl6, Palld, Pld2, Met, Sdc3, Pf4, Sdc4
cell migration	0016477	Nfatc2, Cxcl3, Emp2, Lamc1, Eng, Fscn1, Jup, Mmp14, Rab13, 1600029D21Rik, Nrp2 , Nrp1, Axl, Mmp12, Slamf8, Cxcl16, Pdpn, Ccl2, Ccl17, Ccl22, Plekho1, Itgb7, Ext1, Rhoc, Acvr1, Cd2ap, Nav1, Plekhg5, Acvrl1, Tns1, Sema6d, Tubb2a, Nrg1, Gpr183, Cxcl2, Ccl9, Flrt3, Gpr35, Vav2, Cx3cl1, C230081A13Rik, Tesk1, Megf8, Ccl7, Pmp22, Ccr7, Lgals3, Ccr5, Mertk, Ptpro, Fyn, Amica1, Mcoln2, Palld, Met, Pf4, Sdc3, Sdc4
locomotion	0040011	Nfatc2, Cxcl3, Lamc1, Emp2, Eng, Fscn1, Jup, Rab13, Mmp14, 1600029D21Rik, Nrp2, Nrp1, Mmp12, Axl, Slamf8, Cxcl16, Pdpn, Ccl2, Ccl17, Ccl22, Plekho1, Itgb7, Ext1, Pdgfa, Ch25h, Rhoc, Acvr1, Cd2ap, Nav1, Acvrl1, Plekhg5, Tns1, Sema6d, Tubb2a, Nrg1, Gpr183, Cxcl2, Cttn, Ccl9, Flrt3, Gpr35, Vav2, C230081A13Rik, Cx3cl1, Megf8, Tesk1, Ccl7, Cysltr1, Pmp22, Ccr7, Lgals3, Ccr5, Mertk, Ptpro, Fyn, Amica1, Mcoln2, Insl6 , Palld, Pld2, Met, Cmtm3, Pf4, Sdc3, Sdc4
lymphocyte migration	0072676	Ccr7, Ccl2, Ccl17, Ch25h, Ccl9, Ccl22, Cx3cl1, Gpr183, Itgb7, Cxcl16, Ext1, Ccl7
movement of cell or subcellular component	0006928	Nfatc2 , Cxcl3 , Emp2, Lamc1, Eng, Fscn1, Jup, Mmp14, Rab13, 1600029D21Rik, Nrp2, Nrp1, Mmp12, Uchl1, Axl, Slamf8, Cxcl16, Pdpn, Ccl2, Ccl17, Ccl22, Plekho1, Itgb7, Ext1, Ch25h, Rhoc, Acvr1, Cd2ap, Nav1, Acvrl1, Plekhg5, Dst, Ift43, Tns1, Sema6d, Tubb2a, Nrg1, Gpr183, Nr4a3, Cxcl2, Cttn, Ccl9, Flrt3, Gpr35, Plxna1, Vav2, C230081A13Rik, Evl, Cx3cl1, Megf8, Tesk1, Ccl7, Pmp22, Ccr7, Lgals3, Ccr5, Mertk, Ptpro, Fyn, Amica1, Mcoln2, Insl6, Dpysl2, Palld, Pld2, Met, Pf4, Rasgrp1, Sdc3, Sdc4
lymphocyte chemotaxis	0048247	Ccr7, Ccl2, Ccl17, Ch25h, Ccl22, Cx3cl1, Cxcl16, Ccl7
chemotaxis	0006935	Sema6d, Cxcl3, Nrg1, Gpr183, Eng, Cxcl2, Ccl9, Gpr35, Rab13, Nrp2, Cx3cl1, Slamf8, Cxcl16, Ccl7, Cysltr1, Ccl2, Ccr7, Ccl17, Lgals3, Ccr5, Ccl22, Ptpro, Pdgfa, Amica1, Ch25h, Plekhg5, Cmtm3, Pf4
taxis	0042330	Sema6d, Cxcl3, Nrg1, Gpr183, Eng, Cxcl2, Ccl9, Gpr35, Nrp2, Cx3cl1, Slamf8, Cxcl16, Ccl7, Cysltr1, Ccl2, Ccr7,

		Ccl17, Ccr5, Lgals3, Ccl22, Ptpro, Pdgfa, Amica1, Ch25h, Plekhg5, Cmtm3, Pf4
cell chemotaxis	0060326	Ccr7 , Ccl2 , Ccr5, Lgals3, Ccl17, Cxcl3, Ccl22, Gpr183, Ptpro, Eng, Cxcl2 , Amica1, Ch25h, Ccl9, Gpr35, Rab13, Cx3cl1, Slamf8, Cxcl16, Plekhg5, Ccl7, Pf4
monocyte chemotaxis	0002548	Ccl2, Lgals3, Ccl17, Ccl22, Ccl9, Cx3cl1, Ptpro, Ccl7
leukocyte chemotaxis	0030595	Ccl2, Ccr7, Ccl17, Ch25h, Ccl22, Cxcl3, Cx3cl1, Ptpro, Slamf8, Cxcl16, Ccl7, Pf4
leukocyte migration	0050900	Ccr7, Ccl2, Lgals3, Ccl17, Cxcl3, Ccl22, Gpr183, Ptpro, Itgb7, Ext1, Cxcl2, Amica1, Ch25h, Ccl9, Mcoln2, Gpr35, Cx3cl1, Slamf8, Cxcl16, Ccl7, Pf4
mononuclear cell migration	0071674	Ccl2, Ccl17, Lgals3, Amica1, Ccl9, Ccl22 , Cx3cl1, Ptpro, Ccl7
myeloid leukocyte migration	0097529	Ccl2, Ccr7, Ccl17, Ccl22, Cxcl3, Mcoln2, Cx3cl1, Ptpro, Ccl7, Pf4
**2.) cell adhesion**		
**biological process**	**GO ID**	**linked target genes**
biological adhesion	0022610	Bcl2, Emp2, Lamc1, Tgfbi, Gbp3, Itga1, Eng, Jup, Stab1, Flrt3, Plxnb2, Adam23, C230081A13Rik, Cx3cl1, Nrp1, Axl, Igsf9, Mllt4, Tjp2, Serpinb8, Pdpn, Gpnmb, Cd6, Pmp22, Itgax, Lgals1, Mpzl3, Cdh1, Itgae, Mertk, Inppl1, Itgb7, Ext1, Cd200r1, Gbp2, Fblim1, Cd36, Amica1, Acvr1 , Cd2ap, Palld, Dst, Parvb, Sdc3, Mfge8
cell adhesion	0007155	Bcl2, Emp2, Lamc1, Tgfbi, Itga1, Eng , Jup, Stab1, Plxnb2, Flrt3, Adam23, Cx3cl1 , C230081A13Rik, Nrp1, Axl , Igsf9, Mllt4, Tjp2, Serpinb8, Pdpn, Cd6, Gpnmb, Pmp22, Itgax, Lgals1, Mpzl3, Cdh1, Itgae, Mertk, Inppl1, Itgb7, Ext1, Cd200r1, Fblim1, Cd36, Amica1, Acvr1, Cd2ap, Palld, Dst, Parvb, Sdc3, Mfge8
positive regulation of cell-cell adhesion	0022409	Ccl2, Pdcd1lg2, Ccr7, Il7r, H2-Ab1, H2-Aa, Aif1, Nr4a3, Cd74, Cd86, Cd83, Il2ra, Cx3cl1, Pdpn , Rasgrp1, Cd6
positive regulation of cell adhesion	0045785	Ccr7, Ccl2, Pdcd1lg2, Il7r, H2-Ab1, H2-Aa, Aif1, Emp2, Nr4a3, Cd74, Cd86, Cd36, Cd83, Il2ra, Cx3cl1, Pdpn, Cd6, Sdc4
regulation of leukocyte cell-cell adhesion	1903037	Ccl2, Ccr7, Pdcd1lg2, Il7r, H2-Ab1, Aif1, H2-Aa, Nr4a3, Cd74, Cd86, Cd83, Il2ra, Rasgrp1, Cd6, Sdc4
regulation of cell-cell adhesion	0022407	Aif1, Il1a, Trpv4, Nr4a3, Cd74, Myo10, Cd86, Zfp703, Cd83, Cx3cl1, Cd244, H2-M3, H2-DMa, Mllt4, Pdpn, Kifap3, Gpnmb, Cd6, Shb , Il6st, Pdcd1lg2, Ccr7, Ccl2, Lgals3, Ccr5, Klf4, Lgals1, Il7r, Cdh1, H2-Ab1, H2-Aa, Malt1, Hfe, Ass1, Tnfsf9, Il2ra, Cd1d1, Rasgrp1, Sdc4
**3.) leukocyte activation and immune response**		
**biological process**	**GO ID**	**linked target genes**
immune response	0006955	Cxcl3, Emp2, Il1a, Rnase6, Cd74, Cd86, Slamf7, Axl, Traf3ip2, H2-DMa, Cd6, H2-DMb1, Serpinb9, Tnfrsf11a, Ccl2, Ccl17, Ccl22, Ext1, Tnfsf12, Mfhas1, Tnfsf8, Cd36,

		Cd1d1, Gpr183, Trem2, Cxcl2, Lta, Ptx3, Ccl9, Cx3cl1, Cd244, Irg1, Ccl7, Ctss, Pmp22, Cysltr1, Ccr7, Pdcd1lg2, Prdx1, Ccr5, Lgals3, Tmem106a, H2-Ab1, beta 1, H2-Aa, C1qc, Wfdc17, C1qb, H2-Eb1, Fyn, Mcoln2, Ly86, Il2ra, Rasgrp1, Pf4, Lat2
lymphocyte migration	0072676	Ccr7, Ccl2, Ccl17, Ch25h, Ccl9, Ccl22, Cx3cl1, Gpr183, Itgb7, Cxcl16, Ext1, Ccl7
positive regulation of immune system process	0002684	Bcl2, Nfatc2, Aif1, Gbp5, Il1a, Cd74, Cd86, Cd83, Mmp14, Pgf, Axl, Mmp12, Dcstamp, Car2, H2-DMa, Cd6, Shb, Ccl2, Cd40, Mfhas1, Cd36, Rftn1, Cacnb3, Cd1d1, Tnip3, Scimp, Stk39, Blnk, Gpr183, Trem2, Trpv4, Nr4a3, Lta, Lgmn, Cx3cl1, Cd244, Irg1, Gpr68, Pdcd1lg2, Il6st, Ccr7, Lgals3, Il7r, H2-Ab1, C1qc, H2-Aa, Pyhin1, C1qb, Fyn, Il2ra, Pld2, Cmtm3, BC096441, Nlrc5, Kcnn4, Pf4, Rasgrp1, Lat2, Zbtb46
lymphocyte chemotaxis	0048247	Ccr7, Ccl2, Ccl17, Ch25h, Ccl22, Cx3cl1, Cxcl16, Ccl7
monocyte chemotaxis	0002548	Ccl2, Lgals3, Ccl17, Ccl22, Ccl9, Cx3cl1, Ptpro, Ccl7
leukocyte chemotaxis	0030595	Ccl2, Ccr7, Ccl17, Ch25h, Ccl22, Cxcl3, Cx3cl1, Ptpro, Slamf8, Cxcl16, Ccl7, Pf4
positive regulation of lymphocyte activation	0051251	Bcl2, Nfatc2, Aif1, Gpr183, Il1a, Cd74, Cd86, Cd83, Mmp14, Axl, Cd244, H2-M3, H2-DMa, Cd6, Shb, Ccl2, Il6st, Ccr7, Pdcd1lg2, Lgals1, Il7r, H2-Ab1, H2-Aa, Malt1, Cd40, Tlr9, Tnfsf9, Il2ra, Cd1d1, BC096441, Rasgrp1
positive regulation of leukocyte activation	0002696	Bcl2, Nfatc2, Aif1, Gpr183, Il1a, Trem2, Nr4a3, Cd74, CD86, Mmp14, Cd83, Axl, Cd244, H2-M3, H2-DMa, Cd6, Shb, Ccl2, Pdcd1lg2, Ccr7, Il6st, Lgals1, Il7r, H2-Ab1, H2-Aa, Malt1, Cd40, Tlr9, Tnfsf9, Il2ra, Cd1d1, Pld2, BC096441, Rasgrp1
leukocyte migration	0050900	Ccr7, Ccl2, Lgals3, Ccl17, Cxcl3, Ccl22, Gpr183, Ptpro, Itgb7, Ext1, Cxcl2, Amica1, Ch25h, Ccl9, Mcoln2, Gpr35, Cx3cl1, Slamf8, Cxcl16, Ccl7, Pf4
defense response	0006952	Cxcl3, Gbp5, Spire1, Il1a, Rnase6, Cd74, Cd86, Slamf7, Axl, Slamf8, Traf3ip2, Cd6 , Serpinb9, Itgax, Ccl2, Ccl17, Ccl22, Cd40, Ext1, Gbp2, Mfhas1, Tnfsf8, Cd36, Acvr1, Cd1d1, Gbp3, Cst3, Trem2, Cxcl2, Lta, Ptx3 , Ccl9 , Cx3cl1, Cd244, Irg1, Ccl7, Cysltr1, Ccr7, Adra2a, Lgals3, Ccr5, Prdx1, Il7r, Tmem106a, C1qc, Ciita, Wfdc17, C1qb, Batf2, Fyn, Mcoln2, Ly86, Il2ra, Pf4, Rasgrp1
antigen processing and presentation of exogenous peptide antigen via MHC class II	0019886	Cd74, H2-Ab1, H2-Aa, H2-DMa, H2-DMb2, Ctss, H2-Eb1, H2-DMb1, Ifi30
antigen processing and presentation of peptide antigen via MHC class II	0002495	Cd74, H2-Ab1, H2-Aa, H2-DMa, H2-DMb2, Ifi30, H2-DMb1, H2-Eb1, Ctss

antigen processing and presentation of peptide or polysaccharide antigen via MHC class II	0002504	Cd74, H2-Ab1, H2-Aa, H2-DMa, H2-DMb2, Ctss, H2-Eb1, H2-DMb1, Ifi30
positive regulation of leukocyte cell-cell adhesion	1903039	Ccl2, Pdcd1lg2, Ccr7, Il7r, H2-Ab1, Aif1, H2-Aa, Nr4a3, Cd74, Cd86, Cd83, Il2ra, Rasgrp1, Cd6
negative regulation of immune system process	0002683	Pdcd1lg2, Il7r, Susd4, H2-Ab1, C1qc, H2-Aa, Mfhas1, H2-Ob, Cd74, Cd86, Il2ra, Cx3cl1, Klrb1b, Axl, Mmp12, Slamf8, Pf4, Gpnmb, Sdc4, Zbtb46, Serpinb9
positive regulation of T cell activation	0050870	Ccl2, Pdcd1lg2, Ccr7, Il7r, H2-Ab1, Aif1, H2-Aa, Cd74, Cd86, Cd83, Il2ra, Cd6, Rasgrp1
mononuclear cell migration	0071674	Ccl2, Ccl17, Lgals3, Amica1, Ccl9, Ccl22, Cx3cl1, Ptpro, Ccl7
adaptive immune response	0002250	Tnfrsf11a, Pdcd1lg2, Cd74, Fyn, Cd86, Mcoln2, H2-Ab1, H2-Aa, Emp2, Slamf7, H2-Eb1, Serpinb9
humoral immune response	0006959	Ccl2, Lgals3, Ccl17, Cxcl3, Ccl22, H2-Ab1, C1qc, Gpr183, Wfdc17, C1qb, Rnase6, Cxcl2, Lta, Traf3ip2, Pf4
regulation of leukocyte cell-cell adhesion	1903037	Ccl2, Ccr7, Pdcd1lg2, Il7r, H2-Ab1, Aif1, H2-Aa, Nr4a3, Cd74, Cd86, Cd83, Il2ra, Rasgrp1, Cd6, Sdc4
positive regulation of leukocyte differentiation	1902107	Ccr7, Il7r, H2-Aa, Malt1, Trem2, Il1a, Cd74, Tnfsf9, Cd83, Mmp14, Il2ra, Axl, Cd1d1, Dcstamp, H2-M3, Car2, H2-DMa, Rasgrp1, Pf4, Gpr68, Zbtb46, Shb
myeloid leukocyte migration	0097529	Ccl2, Ccr7, Ccl17, Ccl22, Cxcl3, Mcoln2, Cx3cl1, Ptpro, Ccl7, Pf4
inflammatory response	0006954	Cxcl3, Gbp5, Trem2, Il1a, Cd180, Cxcl2, Lta, Ccl9, Stab1, Cx3cl1, Axl, Irg1, Traf3ip2, Ccl7, Cd6, Cysltr1, Serpinb9, Ccr7, Ccl2, Adra2a, Ccr5, Ccl17, Ccl22, Ciita, Cd40, Ext1, Mfhas1, Tlr9, Ly86, Il2ra, Acvr1, Pld3, Pld4, Rasgrp1, Pf4
regulation of leukocyte differentiation	1902105	Ccr7, Cd74, Il7r, Cd83, H2-Aa, C1qc, Il2ra, Dcstamp, Slamf8, Rasgrp1, Pf4, Zbtb46
antigen processing and presentation of exogenous peptide antigen	0002478	Cd74, H2-Ab1, H2-Aa, H2-DMa, H2-DMb2, Ctss, H2-DMb1, H2-Eb1, Ifi30
myeloid leukocyte migration	0097529	Ccl2, Ccr7, Ccl17, Ccl22, Cxcl3, Mcoln2, Cx3cl1, Ptpro, Ccl7, Pf4
**4.) ERK1 and ERK2 cascade**		
**biological process**	**GO ID**	**linked target genes**
regulation of ERK1 and ERK2 cascade	0070372	Ccl2, Tnfrsf11a, Ccl17, Ccl22, Pdgfa, Trpv4, Mfhas1, Cd74, Cd36, Tnfaip8l3, Cx3cl1, Ramp3, Scimp, Ccl7, Rasgrp1, Gpnmb
positive regulation of ERK1 and ERK2 cascade	0070374	Ccl2, Tnfrsf11a, Ccl17, Ccl22, Pdgfa, Trpv4, Mfhas1, Cd74, Cd36, Tnfaip8l3, Cx3cl1, Ramp3, Scimp, Ccl7, Rasgrp1, Gpnmb

5.) response to cytokine		
**biological process**	**GO ID**	**linked target genes**
chemokine-mediated signaling pathway	0070098	Cxcl2, Ccl2, Stk39, Ccl17, Ccl9, Ccl22, Cxcl3, Gpr35, Ccl7, Pf4
response to interferon-gamma	0034341	Ccl2, Ccl17, Mrc1, Ccl22, H2-Ab1, H2-Aa, Cd40, Ciita, Gbp5, Gbp3, Gbp2, Kynu, H2-Eb1, Cd74, Ccl9, Evl, Cx3cl1, Cxcl16, Irg1, Ccl7
**6.) response to stress**		
**biological process**	**GO ID**	**linked target genes**
response to tumor necrosis factor	0034612	Tnfrsf11a, Ccl2, Ccl17, Ccl22, Cx3cl1, Cd40, Dcstamp, Cxcl16, Ccl7

Suppl. Tab. S5: GO terms of biological processes and linked target genes down-regulatedin CD11b+CD11c+ MDSCs compared to CD11b+CD11c- MDSCs

**Immune and defense response**		
**biological process**	**GO ID**	**linked target genes**
defense response	0006952	Fcnb, Ngp, Gal, Lbp, Cd177, Itgb2l, Camp, Pglyrp1, Elane, S100a8, S100a9, Mpo, Serpinb1a, Cd55, Ear6, Ltf, Chi3l3, Ctsg, Lcn2, Plac8
defense response to fungus	0050832	Mpo, Ltf, Ctsg, Camp, Elane
defense response to bacterium	0042742	Mpo, Lbp, Ltf, Ctsg, Camp, Pglyrp1, Elane, Lcn2, Plac8
antifungal humoral response	0019732	Ltf, Camp
response to bacterium	0009617	Mpo, Slfn4, Lbp, Ltf, Ctsg, Camp, Pglyrp1, Elane, Lcn2, Lrg1, Plac8
response to fungus	0009620	Mpo, Ltf, Ctsg, Camp, Elane
disruption of cells of other organism	0044364	Ltf, Ctsg, Pglyrp, Camp, Elane, Trem3
killing of cells of other organism	0031640	Ltf, Ctsg, Pglyrp1, Camp, Elane, Trem3,
regulation of inflammatory response	0050727	Ets1, Pparg, Lbp, Nt5e, Cst7, Il17ra, Mefv, Il16, Fpr2, Cd55, Calcrl, C3, Pde2a, Alox5, Aldh2, Abcd2, Pglyrp1, S100a8, Bcr, S100a9, Il1r2, Tgm2, Ffar2, Cd47, Siglece, Ptges, Ager, Siglecg
organ or tissue specific immune response	0002251	Ifnlr1, Ffar2, Ear6, Ltf, Camp
mucosal immune response	0002385	Ifnlr1, Ffar2, Ear6, Ltf, Camp
innate immune response in mucosa	0002227	Ear6, Ltf, Camp
regulation of defense response	0031347	Ifnlr1, Ets1, Lbp, Pparg, Cst7, Nt5e, Nod1, Mefv, Il17ra, Cd37, Il16, Fpr2, Il27, Mmp2, Cd55, Ltf, Calcrl, Ceacam1, Irf7, C3, Pde2a, Fcnb, Alox5, Aldh2, Abcd2, Pglyrp1, S100a8, Trem3, S100a9, Bcr, Il1r2, Tgm2, Flot1, Ffar2, Gfi1, Serpinb1a, Tspan32, Cd47, Ptges, Siglece, Ager, Siglecg
humoral immune response	0006959	Fcnb, CD55, Ltf Ctsg, Pglyrp1, Camp, S100a9
